# Genetic structure of two *Prosopis* species in Chaco areas: A lack of allelic diversity diagnosis and insights into the allelic conservation of the affected species

**DOI:** 10.1002/ece3.4137

**Published:** 2018-06-07

**Authors:** Fábio M. Alves, Ângela L. B. Sartori, Maria I. Zucchi, Ana M. G. Azevedo‐Tozzi, Evandro V. Tambarussi, Alessandro Alves‐Pereira, Anete P. de Souza

**Affiliations:** ^1^ Department of Plant Biology Institute of Biology University of Campinas – UNICAMP Campinas SP Brazil; ^2^ Center for Molecular Biology and Genetic Engineering (CBMEG) University of Campinas – UNICAMP Campinas SP Brazil; ^3^ Center of Biological and Health Sciences (CCBS) Federal University of Mato Grosso do Sul – UFMS Campo Grande MS Brazil; ^4^ São Paulo Agency of Technology and Agro‐Business Piracicaba SP Brazil; ^5^ Department of Forestry Engineering University of the Midwest Irati PR Brazil

**Keywords:** conservation genetics, population genetics, *Prosopis rubriflora*, *Prosopis ruscifolia*, South Pantanal

## Abstract

The Gran Chaco is the largest continuous region of the South American dry forest, spanning Argentina, Paraguay, Bolivia, and Brazil. *Prosopis rubriflora* and *Prosopis ruscifolia* are typical tree species of chaquenian area forests, which have been subjected to continuous fragmentation caused by cattle raising. This study evaluated *P. rubriflora* and *P. ruscifolia* in areas with varying levels of disturbance. We investigated the contemporary genetic diversities of both species in areas with distinct anthropogenic disturbances. Even with a lower heterozygote frequency, disturbed areas can provide important storage for alleles, allowing the maintenance of diversity. The genetic diversity of *P. rubriflora* was surprisingly similar to that of *P. ruscifolia* (*H*_e _= 0.59 and *H*_e_ = 0.60, respectively) even with very different distribution ranges of both species. However, *P. ruscifolia* exhibited a higher intrapopulation fixation index than *P. rubriflora*. *P. rubriflora* showed evidence of bottlenecking in 64% of the sampled areas, while *P. ruscifolia* showed such evidence in 36% of the sampled areas. Additionally, *P. rubriflora* had two distinct populations due to its disjunctive geographic distribution, whereas *P. ruscifolia* had a single population that exhibited few signs of population structure in some areas, possibly due to the main pollinators presenting a short range of dispersion. Our results suggest that 42 Chaco areas should be conserved to retain the minimum of 500 individuals necessary to maintain genetic diversity for 100–1,000 generations. This study improves our understanding of these two *Prosopis* species and provides information for the conservation of their genetic diversities.

## 1 INTRODUCTION

The size of the Gran Chaco is approximately 800,000 km²; this area represents the largest dry forest in South America and spans Argentina, Paraguay, Bolivia, and Brazil (Hueck, 1972). The Brazilian areas with chaquenian influence are located mainly in the South Pantanal region (wetland) within the Nabileque subregion and Porto Murtinho in Mato Grosso do Sul County (Silva & Abdon, 1998) and cover 12,419 km² according to the RADAM (*Radar da Amazônia*) Brazil project (Furtado, Guimarães, & Fonzar, 1982; Loureiro, Lima, & Fonzar, 1982). As the northeast border of the Gran Chaco is located in the South Pantanal, this region also shares some floristic elements of other phytophysiognomies, such as the Cerrado and deciduous forests; therefore, to locate characteristics elements of the chaquenian flora, the use of key species, such as members of the genus *Prosopis* L., is important for identifying which areas are considered truly chaquenian.

The genus *Prosopis* has ecological and economic importance in arid regions (Burkart, 1976; Shackleton, Le Maitre, Pasiecznik, & Richardson, 2014) and is associated with chaquenian areas (Prado, 1993). The genus is mainly pollinated by insects (Bessega, Pometti, Ewens, Saidman, & Vilardi, 2012; Bessega et al., 2000; Burkart, 1976), which present a short distance of pollen dispersion (Bessega et al., 2000, 2012). The mating system of *Prosopis* varies from allogamous, such as *P. alba* Griseb. (Bessega et al., 2012), *P. glandulosa* Torr. and *P. nigra* (Griseb.) Hieron (Bessega et al., 2000), to mixed mating systems, such as *P. ruscifolia* and *P. velutina* (Wooton) Sarg. (Bessega et al., 2000). The seed dispersion of *Prosopis* is zoochoric (Burkart, 1976; Solbrig & Cantino, 1975) and depends on domesticated animals, such as cattle, goats, and horses, and wild animals, such as armadillos, foxes, skunks, and rodents (Burkart, 1976; Campos, Campos, Miguel, & Cona, 2016). The genus also presents an autochoric dispersion because the mature fruits undergo abscission of the pedicel, and causing them to fall below the matrix trees (Freitas et al., 2013; Solbrig & Cantino, 1975).

*Prosopis rubriflora* is a tree species with a height range of 5–6 m, and its branches are armed with prickles, red inflorescences, and reduced linear leaflets (Burkart, 1976). This species has two flowering peaks, with the first occurring in February and the second occurring in August. Maximum fruiting occurs from October to January, but this species continuously flowers throughout the year at a lower intensity (Stefanello, 2012). *Prosopis rubriflora* is associated with “arborized stepic savanna,” which primarily consists of sparse nanophanerophytes (IBGE, 2012). This species is often found in clusters interspersed with other species that are commonly dominant in conserved areas (Lima, 2012). *Prosopis rubriflora* is observed in the southern region in Mato Grosso do Sul, Brazil, and in northeastern Paraguay (Burkart, 1976) frequently associated with the chaquenian areas in Brazil (Pott & Pott, 1994) with arboreal physiognomy. This tree has ornamental potential and is recommended for landscape design, as the wood can be used as charcoal and agricultural instruments (Lorenzi, 2002). This species was considered endangered in Paraguay according to the IUCN 1997 list (Walter & Gillett, 1998).

*Prosopis ruscifolia* is a tree species with a height range of 5–12 m, and its branches are armed with white inflorescences and large oval‐lanceolate leaflets (Burkart, 1976). This species annually produces flowers and fruits (November to February) and is typically found in forested stepic savanna, which consists of micro‐ and/or nanophanerophytes of varying densities. A woody, grassy layer is less common (IBGE, 2012). *Prosopis ruscifolia* commonly borders conserved and disturbed forested areas, and this species has larger distributions in the chaquenian areas of Bolivia, Paraguay, and Argentina (Burkart, 1976), which are associated with the chaquenian areas of Brazil (Pott & Pott, 1994). The wood can be used for making furniture and frames, firewood, activated carbon, and external uses, such as posts. The pods produced are edible and can be processed into flour or cooked; in addition, they are fodder for livestock (Lorenzi, 2002). The tree has been indicated for the reforestation of degraded riparian areas and is considered a valuable species for forest restoration in semi‐arid chaquenian areas (Blasco, Astrada, & Carenzo, 2006).

Although the Chaco areas in Porto Murtinho are considered to have biological importance (Ministério do Meio Ambiente, 2002), no Brazilian chaquenian areas are currently designated as conservation areas (Pott & Pott, 2003) to ensure minimal genetic diversity of native species. According to data from MMA‐IBAMA (2010), the Pantanal areas of Porto Murtinho, the location of the majority of Brazil’s chaquenian areas, lost 35.81% of their native coverage by 2008.

Habitat fragmentation reduces the number of individuals and the genetic diversity of the population. Thus, it may reduce the effective population size (*N*_e_) to a level that causes genetic drift in the short term due to the loss of rare alleles (*A*_r_). Habitat fragmentation may cause inbreeding in the long term due to an increased probability of pollination between related and inbred individuals (Kageyama & Gandara, 1998). The allele frequency in large populations tends to be reduced by genetic drift; however, this effect is stronger in smaller populations in which genetic drift causes allele frequencies to change randomly, leading to the loss or fixation of alleles over time due to the limited alleles presented in the parental generation (Ellstrand & Elam, 1993). Furthermore, these new allele frequencies contribute to increased differentiation among populations. Smaller populations trend toward increased individual homozygosity and inbreeding depression, which tends to reduce fitness and affect the fertility and survival of individuals (Charlesworth & Willis, 2009), especially for noninbred species (Ellstrand & Elam, 1993).

In this context, this study aimed to test the following hypotheses: (i) the genetic diversity of *P. rubriflora* should be lower than that of *P. ruscifolia* due to the smaller geographic distribution of the former, and (ii) strong genetic structures are present in both species due to the behavior of the main pollinating agent, as described in the literature on *Prosopis*. Thus, the objectives of this study were to evaluate the genetic diversity and to estimate the genetic structures of the sampled areas containing *P. rubriflora* and *P. ruscifolia* to examine whether they have been influenced by distinct anthropogenic disturbances.

## 2 MATERIALS AND METHODS

### 2.1 Plant materials

**Figure 1:**
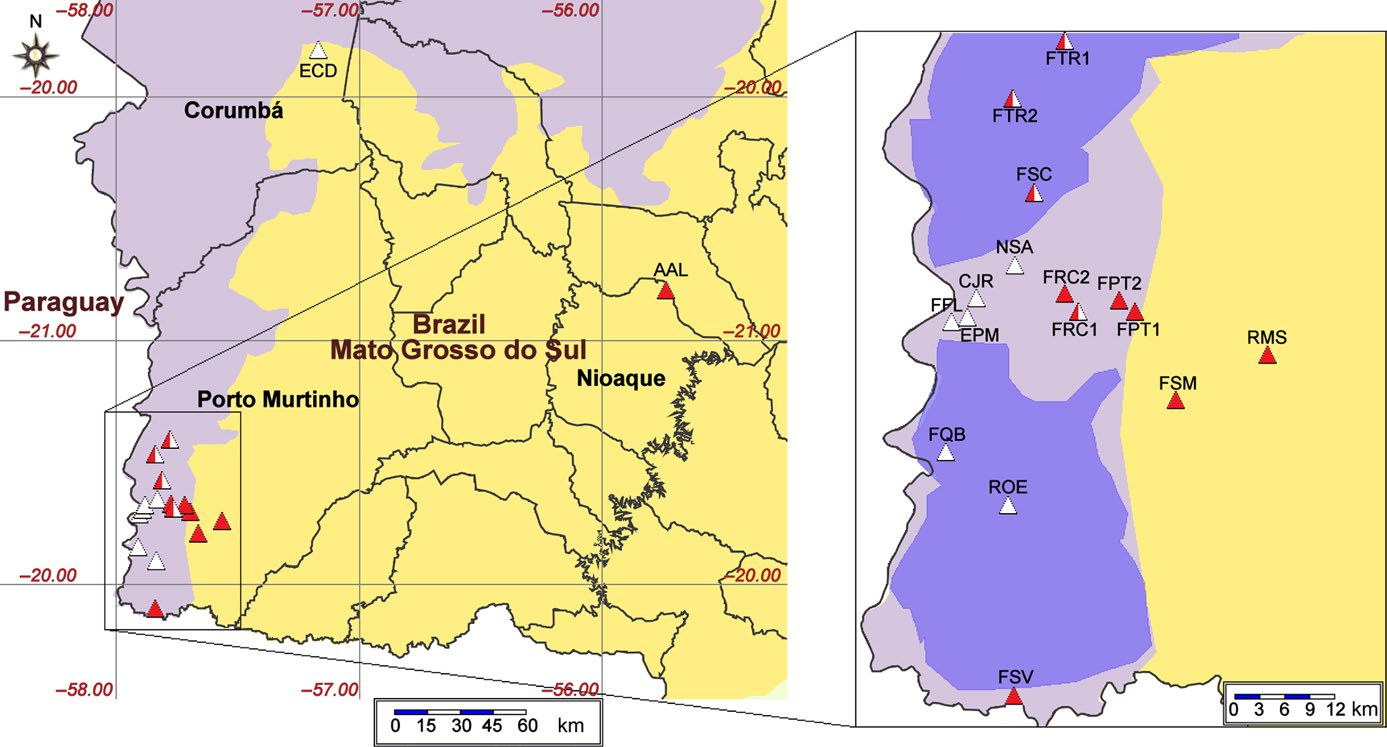
Locations at which samples of *Prosopis rubriflora* and *Prosopis ruscifolia* were collected. The sampled areas are represented by triangles: red indicates *P. rubriflora*, white indicates *P. ruscifolia*, and the combination of red and white indicates areas where both taxa were collected. On the map, the yellow areas represent the Cerrado domain, the purple areas represent the Pantanal domain, and the dark purple areas represent the priority areas for conservation for the Pantanal domain. The sampled area codes are presented in Table 1. The map was created by the speciesMapper tool, which is available from the *species*Link project (http://splink.cria.org.br/tools)

We considered 19 chaquenian areas, also known as stepic savanna, from which samples of *P. rubriflora* and *P. ruscifolia* were collected in the Corumbá, Nioaque, and Porto Murtinho counties of the State of Mato Grosso do Sul, Brazil (Figure 1). Stepic savanna is composed of neotropical steppe vegetation cover with low and spiny plants in a grassy savanna (Furtado et al., 1982). The climate is hot and dry, with a reduced annual rainfall of approximately 1,000 mm (Loureiro et al., 1982). Seasonal rains are concentrated from November to February (rainfall ≥100 mm), and a drought occurs from June to September during the dry season (Stefanello, 2012). The soils are saline with limited drainage due to the *fragipan* horizon, and the land is covered in water for several months. When the soil is drained, it becomes parched (Furtado et al., 1982).

The sampled areas were classified according to the observed levels of anthropogenic disturbance based on field observations and surveys by the farm owners. “Conserved areas” represent no to low disturbance levels, such as cattle breeding activity without any evidence of anthropogenic suppression. The “intermediate disturbance areas” have experienced recent suppression in vegetation (10–15 years) or have a predominance of colonizing species, such as *Parkinsonia praecox* and *Mimosa hexandra* Micheli. The “degraded areas” show a predominance of pastures or land in the initial stage of regeneration of woody plants. The coordinates were obtained from a global positioning system (GPS) using WGS‐84 datum.

Both species were sampled in the following areas of Porto Murtinho: Fazenda Retiro Conceição (area 1), Fazenda Tereré (areas 1 and 2), and Fazenda Santa Cristina, which did not present a continuous conservation status but rather “conserved” fragments surrounded by higher or lower degrees of anthropogenic disturbance. *Prosopis rubriflora* was exclusively located in the following areas: Nioaque County in Assentamento Andalúcia (presenting fragments with lower to intermediate disturbance) and Porto Murtinho County in Fazenda Patolá (areas 1 and 2), Fazenda Retiro Conceição (area 2), Fazenda São Manoel, Fazenda Santa Vergínia, and highway MS‐467, where FPT2 and FRC2 were the only conserved areas, and all the remaining areas presented fragments with higher to lower degrees of disturbance. The remaining areas from which only *P. ruscifolia* was collected were located in Corumbá County (Estação do Carandazal) and Porto Murtinho County (Armed Force Area of Porto Murtinho, Fazenda Quebracho‐Brasil, Retiro Ovo de Ema, Fazenda Flores, Chácara Jacaré, and Fazenda Nossa Senhora Aparecida (Table 1)), presenting areas with high levels of disturbance, such as Estação do Carandazal (ECD) and Fazenda Quebracho‐Brasil (FQB), and the remaining areas presented fragments with “intermediate” levels of disturbance. The voucher specimens were deposited in the Herbarium of Universidade Estadual de Campinas (UEC).

**Table 1:** Sampled areas in which *Prosopis rubriflora* and *Prosopis ruscifolia* were collected and their respective preservation statuses

Taxon	Sampled area (initials)	Preservation	Geographic coordinates
*Prosopis rubriflora*	Assentamento Andalúcia (AAL)	Conserved to intermediate disturbance	55°44′17″W–20°48′05″S
	Fazenda Patolá Area 1 (FPT1)	Intermediate disturbance	57°42′11″W–21°42′09″S
	Fazenda Patolá Area 2 (FPT2)	Conserved	57°43′16″W–21°41′21″S
	Fazenda Santa Cristina (FSC)	Disturbed to conserved	57°48′36″W–21°34′35″S
	Highway MS‐467 (RMS)	Disturbed to intermediate disturbance	57°33′44″W–21°44′57″S
	Fazenda Tereré Area 1 (FTR1)	Conserved to intermediate disturbance	57°46′43″W–21°24′42″S
	Fazenda Tereré Area 2 (FTR2)	Disturbed to conserved	57°49′59″W–21°28′40″S
	Fazenda Retiro Conceição Area 1 (FRC1)	Disturbed to intermediate disturbance	57°45′49″W–21°42′08″S
	Fazenda Retiro Conceição Area 2 (FRC2)	Conserved	57°46′43″W–21°41′05″S
	Fazenda São Manoel (FSM)	Intermediate disturbance	57°39′34″W–21°47′50″S
	Fazenda Santa Vergínia (FSV)	Conserved to intermediate disturbance	57°50′01″W–21°06′42″S
*Prosopis ruscifolia*	Armed Force of Porto Murtinho (EPM)	Intermediate disturbance	57°53′10″W–21°42′31″S
	Fazenda Quebracho‐Brasil (FQB)	Disturbed	57°54′11″W–21°51′06″S
	Retiro Ovo de Ema (ROE)	Intermediate disturbance	57°50′14″W–21°54′34″S
	Fazenda Flores (FFL)	Conserved to intermediate disturbance	57°53′53″W–20°42′52″S
	Chácara Jacaré (CJR)	Intermediate disturbance	57°49′54″W–21°39′17″S
	Fazenda N. Sra. Aparecida (NSA)	Intermediate disturbance	57°49′51″W–21°39′16″S
	Fazenda Santa Cristina (FSC)	Disturbed to conserved	57°48′36″W–21°34′35″S
	Estação do Carandazal (ECD)	Disturbed	57°10′14″W–19°48′34″S
	Fazenda Retiro Conceição Area 1 (FRC1)	Disturbed to intermediate disturbance	57°45′49″W–21°42′08″S
	Fazenda Tereré Area 1 (FTR1)	Conserved to intermediate disturbance	57°46′43″W–21°24′42″S
	Fazenda Tereré Area 2 (FTR2)	Disturbed to conserved	57°49′59″W–21°28′40″S

We collected the leaves and leaflets of 241 *P. rubriflora* individuals and 308 *P. ruscifolia* individuals, ranging from 16 to 30 trees per area (Tables 2 and 3). The distance between the samples ranged from 10 to 1,470 m; however, we aimed to maintain an average distance of 50 m between two individuals to prevent the collection of related samples. The sampled leaves and leaflets that were collected for genomic DNA extraction were initially stored in silica gel and subsequently deposited at −80°C.

**Table 2:** Genetic parameters based on ten microsatellite (SSR) loci analyzed in *Prosopis rubriflora* samples in the 11 areas of arborized stepic savanna

*Prosopis rubriflora*												
Area (preservation)	*N*	*K*	*A*_ri_	*A*_e_ (%)	*A*_p_	*A*_r_	*H*_o_	*H*_e_	*F*_IS_	${\hat{t}}_{a}$	*N*_e_	*N*_e_ */N*
AAL (Con‐Int)	20	41	3.9	23.35 (57%)	1	11	0.40	0.49	**0.17**	0.74	8.91	0.45
FPT1 (Int)	20	57	5.5	31.14 (55%)	1	14	0.57	0.59	0.02	0.87	11.79	0.59
FPT2 (Con)	20	56	5.4	33.54 (60%)	0	15	0.59	0.61	0.03	0.92	11.65	0.58
FSC (Con‐Dis)	25	60	5.4	34.88 (58%)	0	17	0.64	0.61	−0.08	0.96	14.28	0.57
RMS (Int)	27	71	6.1	36.13 (51%)	6	23	0.62	0.64	0.05	0.90	13.12	0.49
FTR1 (Con‐Int)	30	66	5.6	31.33 (47%)	1	23	0.61	0.60	−0.03	0.92	15.10	0.50
FTR2 (Con‐Dis)	16	53	5.3	34.29 (65%)	0	12	0.57	0.60	0.10	0.78	9.19	0.57
FRC1 (Int)	22	61	5.6	30.53 (50%)	0	19	0.53	0.57	0.05	0.86	11.64	0.53
FRC2 (Con)	22	53	5.0	29.08 (55%)	0	12	0.52	0.55	0.09	0.80	11.21	0.51
FSM (Int)	19	58	5.5	32.20 (55%)	1	15	0.55	0.58	0.06	0.87	11.17	0.59
FSV (Con‐Int)	20	64	6.0	35.80 (56%)	2	20	0.58	0.61	0.08	0.84	11.12	0.56
Average	21.91	58.18/9.8^a^	5.39	32.02 (55%)	1.09	16.45	0.56	0.59	0.05	0.86	11.74	0.54

The sampled area codes are presented in Table 1. Preservation status: Con, conserved; Dis, disturbed; Int, intermediate disturbance *N*, number of sampled individuals in the sampled areas; *k*, number of alleles; *A*_ri_, allelic richness; *A*_e_, effective number of alleles and respective percentage; *A*_p_, private alleles; *A*_r_, rare alleles; *H*_o_, observed heterozygosity; *H*_e_, expected heterozygosity; *F*_IS_, Wright fixation index; ${\hat{t}}_{a}$, apparent outcrossing rate; *N*_e_, effective number; *N*_e_ */N*, genetic representativeness of *N*_e_. The bold values indicate areas in which the fixation index significantly differed from zero according to the *p‐*value, with adjusted nominal values of 5% and 110,000 permutations determined using FSTAT 2.9.3.2 software.

a The bold value was indicated in highlight for the Fis analysis for the AAL area.

**Table 3:** Genetic parameters based on 11 microsatellite (SSR) loci analyzed for *Prosopis ruscifolia* samples in the 11 forested stepic savanna areas

*Prosopis ruscifolia*												
Area (preservation)	*N*	*K*	*A*_ri_	*A*_e_	*A*_p_	*A*_r_	*H*_o_	*H*_e_	*F*_IS_	${\hat{t}}_{a}$	*N*_e_	*N*_e_ */N*
EPM (Int)	20	50	4.4	30.51 (61%)	1	8	0.46	0.54	**0.16**	0.76	8.81	0.44
FQB (Dis)	30	80	6.6	46.86 (59%)	7	25	0.56	0.64	**0.17**	0.70	11.84	0.39
ROE (Int)	30	67	5.4	36.37 (54%)	2	21	0.60	0.63	0.01	0.86	14.22	0.47
FFL (Con‐Int)	30	87	6.8	46.99 (54%)	8	32	0.53	0.64	**0.17**	0.71	13.74	0.46
CJR (Int)	30	69	5.5	40.19 (58%)	2	19	0.49	0.59	**0.17**	0.73	11.80	0.40
NSA (Int)	30	68	5.4	35.26 (52%)	0	24	0.56	0.59	0.04	0.86	12.72	0.44
FSC (Con‐Dis)	30	62	5.0	34.30 (55%)	1	16	0.56	0.58	0.01	0.89	14.28	0.49
ECD (Dis)	23	72	6.2	44.19 (61%)	5	25	0.48	0.67	**0.28**	0.57	7.06	0.31
FRC1 (Int)	25	59	5.1	36.67 (62%)	0	13	0.46	0.55	**0.16**	0.75	11.10	0.44
FTR1 (Con‐Int)	30	66	5.4	42.06 (64%)	3	17	0.51	0.59	**0.10**	0.79	13.28	0.46
FTR2 (Con‐Dis)	30	61	5.2	38.70 (63%)	2	14	0.54	0.60	0.06	0.84	13.08	0.44
Average	28.00	67.36/12.54^a^	5.56	39.28 (58%)	2.82	19.45	0.52	0.60	0.12	0.77	11.99	0.43

The sampled area codes are presented in Table 1. Preservation status: Con, conserved; Dis, disturbed; Int, intermediate disturbance. *N*, Number of sampled individuals in the sampled areas; *k*, number of alleles; *A*_ri_, allelic richness; *A*_e_, effective number of alleles and respective percentage; *A*_p_, private alleles; *A*_r_, rare alleles; *H*o, observed heterozygosity; *H*_e_, expected heterozygosity; *F*_IS_, Wright fixation index; ${\hat{t}}_{a}$, apparent outcrossing rate; *N*_e_, effective number; *N*_e_/*N*, genetic representativeness of *N*_e_. The bold values show the areas where the fixation index significantly differed from zero according to the *p*‐value, with adjusted nominal values of 5% and 121,000 permutations determined using FSTAT 2.9.3.2 software.

a Average number of alleles per locus.

### 2.2 DNA extraction, SSR markers, and genotyping procedure

Genomic DNA extraction, fragment amplification, and genotyping were performed according to protocols published by Alves, Zucchi, Azevedo‐Tozzi, Sartori, & Souza (2014). Population genotyping was developed with ten SSR markers for the analysis of *P. rubriflora* (eight specific markers and two markers transferred from *P. ruscifolia*) and 11 SSR markers for the analysis of *P. ruscifolia* (nine specific markers and two markers transferred from *P. rubriflora*) as developed by Alves et al. (2014).

### 2.3 Intrapopulational analysis

The amplified SSR markers for *P. rubriflora* and *P. ruscifolia* from the sampled areas were analyzed for linkage disequilibrium (LD), adherence to Hardy–Weinberg (HW) equilibrium and the frequency of null alleles (NA). LD and HW analyses were performed using Genepop v.1.2 software (Raymond & Rousset, 1995) and Fisher’s exact probability test with 10,000 dememorizations and iterations and 100 batches. The frequency of null alleles was calculated using FreeNA software (Chapuis & Estoup, 2007) assuming that *n* > 0.20 for the presence of NA based on the algorithm of Dempster, Laird, & Rubin (1977).

Allelic frequencies, such as the allele number (*k*), allelic richness (*A*_ri_), *A*_r_, observed heterozygosity (*H*_o_), expected heterozygosity (*H*_e_), and fixation index, and endogamy significance (*F*_IS_), were calculated based on 110,000 permutations for *P. rubriflora* and 121,000 permutations for *P. ruscifolia* with an adjusted nominal level of 5% using the software FSTAT 2.9.3.2 (Goudet, 2001). The confidence intervals (CIs) of *H*_e,_ *H*_o,_ and *F*_IS_ were determined using the diveRsity package (Keenan, McGinnity, Cross, Crozier, & Prodöhl, 2013) for the R software package (R Development Core Team, 2011).

The effective number of alleles (*A*_e_) per locus was estimated according to the equation ${\hat{A}}_{e}=\frac{1}{1-{\hat{H}}_{e}}$ (Jost, 2008; Kimura & Crow, 1964). Wright’s *F* statistic was employed to estimate the population genetic structure by the *F*_IS_, the fixation index between populations (θ or *F*_ST_), and the total endogamy (*F* or *F*_IT_). The number of private alleles (*A*_p_) was estimated with GDA v.1.0 software (Lewis & Zaykin, 2001) using a 95% CI that was determined by bootstrapping with 10,000 replicates.

The apparent outcrossing rate (${\hat{t}}_{a}$), the primary parameter for outcrossing into populations, was obtained according to the equation ${\hat{t}}_{a}=\frac{1-{\hat{F}}_{\mathrm{IS}}}{1+{\hat{F}}_{\mathrm{IS}}}$ as described by Vencovsky (1994). The effective number (*N*_e_) was estimated using an equation derived from Cockerham (1969) that included both the inbreeding index and the average coefficient of coancestry (Θ) from a generation: $N_{e}=\frac{0.5}{\overset{¯}{\theta }\left(\frac{n-1}{n}\right)+\frac{1+F}{2n}}$ (Tambarussi et al., 2016). Kimura and Crow (1963) defined *N*_e_ as the size of an idealized population that would have the same amount of inbreeding or random genetic drift as the same population under consideration. The group coefficient of coancestry for adults within populations was estimated using the coancestry estimated by Loiselle, Sork, Nason, & Graham (1995) implemented in the software SPAGeDi v. 1.3 (Hardy & Vekemans, 2002). The genetic representativeness *m* was estimated as $\frac{N_{e}}{N}$ (Sebbenn, 2003).

### 2.4 Bottleneck

Bottleneck analysis was performed using Bottleneck v.1.2.0 software (Piry, Luikart, & Cornuet, 1999) to identify populations under genetic mutation‐drift equilibrium (simulated coalescent process) as described by Cornuet and Luikart (1996). We employed the stepwise mutation model (SMM) and the two‐phase model (TPM) (12 variations and 95% SMM) with the sign test and the Wilcoxon signed‐rank test (two‐tailed). The sign test utilizes parametric analysis in which the null hypothesis can be rejected based on excess heterozygosity by considering the difference *H*_o_ and *H*_e_ in a significant number of loci in the studied population (Cornuet & Luikart, 1996). The Wilcoxon test compares the observed excess heterozygosity over *H*_e_ to a null hypothesis and is a more robust and sensitive method (Piry et al., 1999).

### 2.5 Global structure and migrants

The historic gene flow (${\hat{N}}_{m}$) was estimated indirectly according to the Crow and Aoki (1984) model as follows: ${\hat{N}}_{m}=\left(\frac{1}{4\alpha }\right)\left[\left(\frac{1}{\theta }\right)-1\right]$, where θ is the divergence index between populations, $\alpha ={\left[\frac{n}{\left(n-1\right)}\right]}^{2}$, and *n* is the number of samples.

Analysis of molecular variance (AMOVA) was performed using the POPR package version 2.5.0 (Kamvar, Brooks, & Grünwald, 2015; Kamvar, Tabima, & Grünwald, 2014), where the *p*‐values were determined after 20,000 replicates. Mantel tests (Mantel, 1967) were performed using the Ade4 package (Dray & Dufour, 2007) to determine correlations between the geographic distance matrix and Nei’s genetic distance (Nei, 1978) with R software (R Development Core Team, 2011). The genetic differentiation between the analyzed areas was estimated by pairwise *F*_ST_ using FreeNA software (Chapuis & Estoup, 2007) with 10,000 iterations. Nei’s genetic distance (Nei, 1978) was estimated to identify similarities or differences between two fragments and complement pairwise *F*_ST_. Nei’s genetic distance analysis was performed using TFPGA v.1.3 software (Miller, 1997) to generate an unweighted pair‐group method of analysis (UPGMA) matrix clustered with 10,000 bootstraps, which provided a basis for the developed dendrogram.

The Bayesian model of the *P. rubriflora* and *P. ruscifolia* genetic structures was developed with Structure v.2.3.2 software (Pritchard, Stephens, & Donnelly, 2000) using admixture and allele frequencies that were correlated between populations. The genetic clusters ranged from *K* = 1 to 15, and each K value was replicated 20 times. The length of the final Markov chain Monte Carlo (MCMC) was 500,000 replicates, with 200,000 replicates for burn‐in. The most likely value of Δ*K* based on Evanno et al. (Evanno, Regnaut, & Goudet, 2005) was estimated using the online tool Clumpak (Kopelman, Mayzel, Jakobsson, Rosenberg, & Mayrose, 2015). Discriminant analysis of principal components (DAPC) analysis was performed using the adegenet package (Jombart, 2008) for R software (R Development Core Team, 2011). Different from Nei’s distance, *F*_ST_, and Bayesian analysis (Structure), DAPC analysis uses a nonparametric approach. The results obtained from this analysis are presented as multidimensional scatterplots of the principal components.

## 3 RESULTS

### 3.1 Intrapopulational evaluation of *Prosopis rubriflora*

Based on the genotyping of *P. rubriflora* individuals, we were able to identify departure from HW equilibrium based on Fisher’s exact test at loci Prb2 and Prb4 and possible null alleles in AAL’s area (Tables S1 and S2). However, no evidence of LD for the evaluated loci was observed after Bonferroni correction (1% and 5% *p*‐values = .0002 and .0011, respectively) (Table S3).

We identified 98 distinct alleles in *P. rubriflora*, with an average of 10 alleles per locus in the 11 sampled areas from the Nioaque and Porto Murtinho counties (Table 2). The number of alleles ranged from 41 for AAL to 71 for RMS and averaged 58 alleles per area. The *A*_ri_ ranged from 3.9 (AAL) to 6.1 (RMS) alleles, and the *A*_e_ ranged from 23 (AAL) to 36 (RMS and FSV) alleles. The percentage of effective alleles relative to the number of sampled alleles varied from 47% (FTR1) to 65% (FTR2). We detected 12 *A*_p_, half of which were detected in the RMS region, followed by FSV (two alleles), FPT1, FTR1, FSM, and AAL (one *A*_p_ per area); the remaining areas had no *A*_p_. Forty‐two *A*_r_, ranging from 11 (AAL) to 23 (RMS and FTR), were detected in the sampled areas, with an average of 16 alleles for all areas. Therefore, *P. rubriflora* contained 45% common alleles, 43% *A*_r,_ and 12% *A*_p_ (Table 2).

The average *H*_o_ was 0.56 and ranged from 0.40 (CI_95%_ = 0.26–0.51) (AAL) to 0.64 (CI_95%_ = 0.47–0.80) (FSC). The average *H*_e_ was 0.59 and ranged from 0.49 (CI_95%_ = 0.34–0.60) (AAL) to 0.64 (CI_95%_ = 0.51–0.73) (RMS) (Table 2). These analyses reveal the current frequency of heterozygotes (*H*_o_) and the expected frequency of heterozygotes (*H*_e_) in a panmictic population according to assumptions of the HW equilibrium model (Frankham, Ballou, & Briscoe, 2008). The *H*_o_ was higher than the *H*_e_ in the areas FSC and FTR1; this difference suggests potential pressure in favor of heterozygotes for these areas compared with the remaining sampled areas.

The average inbreeding coefficient (*F*_IS_) was 0.05 and ranged from −0.08 (CI_95%_ = −0.10 to −0.04) (FSC) to 0.17 (CI_95%_ = 0.08–0.24) (AAL) (Table 2). AAL was the only area with an *F*_IS_ that significantly differed from zero according to the *p‐*value; this difference suggests an excessive quantity of homozygotes in this area compared with the remaining sampled areas (Table S4). The ${\hat{t}}_{a}$ ranged from 0.74 (AAL) to 0.96 (FSC) and averaged 0.86; this result suggests that mixed reproductive systems were functioning in most sampled areas, with a strong allogamous tendency for FSC.

The *N*_e_ parameter suggests that 54% of the genetic contribution arose from individuals in the group of populations (129 of 241 individuals), varying from 45% (AAL) to 59% (FPT1 and FSM) (Table 2).

### 3.2 Intrapopulational evaluation of *Prosopis ruscifolia*

Using the genotyped *P. ruscifolia* samples, we were able to detect departure from HW equilibrium based on Fisher’s exact test for most of the loci, excluding Prb2, Prb4, and Prs3. Possible null alleles were detected in the markers Prs11 (EPM and FRC1) and Prs6 (FTR2) (Tables S5 and S6). After Bonferroni correction (1% *p‐*value = .00018), evidence of LD was noted according to pairwise analysis of the following loci: Prb4 × (Prs11, Prs6, Prs7, Prs1, Prb2, Prs2), Prs7 × (Prs6, Prs1, Prs2, Prb7, Prs11), Prs6 × (Prs1, Prs11), and Prs2 × (Prs1, Prs11) (Table S7). The assumed linkage between these loci was not evident after excluding the following areas: FQB, CJR, NSA, and ECD (Table S8). Therefore, the linkage between the loci may be associated with issues in the areas, such as inbreeding, population subdivision, and potential bottlenecks (Slatkin, 2008). In this context, the markers that revealed deviation in the analysis did not need to be discarded.

In the *P. ruscifolia* dataset, 138 alleles were detected, with an average of 13 alleles per locus for 11 areas in Corumbá (one area) and Porto Murtinho (10 areas). The number of alleles ranged from 4.4 (EPM) to 6.8 (FFL), with an average of 67 alleles per area (Table 3). The *A*_ri_ ranged from 49 (EPM) to 75 (FFL) alleles and averaged 61 alleles. The *A*_e_ ranged from 30 (EPM) to 47 (FFL and FQB) alleles and averaged 39 alleles, producing a variation ranging from 52% (NSA) to 64% (FTR1) compared with the detected allelic number. Additionally, 31 *A*_p_ were detected, with eight *A*_p_ in FFL, seven *A*_p_ in FQB, five *A*_p_ in ECD, three *A*_p_ in FTR1, two *A*_p_ in FTR2 and ROE, one *A*_p_ in EPM and FSC, and no *A*_p_ in the other sampled areas.

*H*_o_ ranged from 0.46 (CI_95%_ = 0.32–0.58) (FRC1 and EPM) to 0.60 (CI_95%_ = 0.50–0.68) (ROE) and averaged 0.52; *H*_e_ ranged from 0.54 (CI_95%_ = 0.42–0.65) (EPM) to 0.67 (CI_95%_ = 0.53–0.76) (ECD) and averaged 0.60. *H*_e_ was greater than *H*_o_, suggesting that the number of homozygotes exceeded the number of heterozygotes in all analyzed areas.

The *F*_IS_ ranged from 0.01 (CI_95%_ = −0.05–0.12 and CI_95%_ = −0.07–0.11, respectively) (FSC and ROE) to 0.28 (CI_95%_ = 0.17–0.37) (ECD) and averaged 0.12. A total of 63% of the areas presented values that significantly differed from zero, suggesting an excessive number of homozygotes based on the *p‐*value (Table S4). The ${\hat{t}}_{a}$ ranged from 0.57 (ECD) to 0.89 (FSC) and averaged 0.77; this result suggests that a mixed reproductive system is present in all areas. The value of *N*_e_ suggested that 43% of all sampled trees provided genic contributions to the sampled areas, and this percentage ranged from 31% in ECD to 49% in FSC (Table 3).

### 3.3 Bottleneck analysis

To reduce the number of errors in the analysis, the markers Prs6 and Prs11 for *P. ruscifolia* were discarded due to their higher proportions of departure from HW equilibrium for most of the evaluated areas (Table S5); according to Luikart and Cornuet (1998), these deviations may generate bias in the results.

Based on the sign test for *P. rubriflora*, the analyzed areas presented five to ten loci with *H*_e_ deficits under mutation‐drift equilibrium (H.d.) and zero to four loci with excess *H*_e_ under mutation‐drift equilibrium (H.e.); the *p*‐values ranged from .000 to .349, as observed in the TPM and SMM results (Table 4). The Wilcoxon signed‐rank test, based on two‐tailed for H.e. and H.d., yielded *p*‐values that ranged from .000 to .375. In *P. ruscifolia*, six to nine loci were under H.d., and zero to four loci were under H.e.; *p*‐values ranged from .004 to .570 according to the sign test with the TPM and SMM. Based on the two‐tailed Wilcoxon analysis for H.e. and H.d., the reported *p*‐values ranged from .000 to .652 for the TPM and SMM (Table 4).

**Table 4:** Bottleneck analysis using the sign and Wilcoxon signed‐rank tests (two‐tailed) for *Prosopis rubriflora* and *Prosopis ruscifolia* sampled in 11 areas

Taxon	Area	Sign test				Wilcoxon test	
		TPM		SMM		TPM	SMM
		H.d./H.e.	*p*	H.d./H.e.	*p*	*p*	*p*
*Prosopis rubriflora*	AAL	05/04	.349	05/04	.325	.250	.164
	FPT1	08/02	**.020**	08/02	**.020**	.084	**.024**
	FPT2	06/04	.221	06/04	.214	.275	.232
	FSC	07/03	.079	07/03	.080	**.024**	**.014**
	RMS	06/04	.192	07/03	.069	.105	**.032**
	FTR1	10/00	**.000**	10/00	**.000**	**.000**	**.000**
	FTR2	06/04	.221	06/04	.213	.375	.232
	FRC1	07/03	.068	08/02	**.015**	.084	**.014**
	FRC2	05/04	.275	05/04	.278	.164	.164
	FSM	07/03	.068	08/02	**.016**	**.014**	**.005**
	FSV	07/03	.069	09/01	**.002**	**.024**	**.009**
*Prosopis ruscifolia*	EPM	06/03	.132	06/03	.137	.496	.300
	FQB	05/04	.310	05/04	.313	.426	.301
	ROE	05/04	.311	05/04	.310	.496	.301
	FFL	08/01	**.004**	08/01	**.004**	**.020**	**.010**
	CJR	05/04	.311	06/03	.125	.301	.250
	NSA	08/01	**.007**	08/01	**.007**	**.027**	**.004**
	FSC	05/04	.311	05/04	.307	.164	.164
	ECD	05/04	.562	05/04	.301	.652	.496
	FRC1	08/01	**.005**	08/01	**.005**	**.020**	**.010**
	FTR1	04/05	.570	06/03	.122	.570	**.049**
	FTR2	05/04	.341	05/04	.340	.652	.426

The sampled area codes for both *P. rubriflora* and *P. ruscifolia* are presented in Table 1. TPM, two‐phase model; SMM, stepwise mutational model; H.d., loci with less *H*_e_; H.e., loci with excess *H*_e_; *p, p*‐value. The bold values represent areas in which the *p*‐value was <.05.

Four sampled areas of *P. rubriflora* (AAL, FPT2, FTR2, and FRC2) and seven areas of *P. ruscifolia* (EPM, FQB, ROE, CJR, FSC, ECD, and FTR2) were reported to be in mutation‐drift equilibrium according to the sign test and Wilcoxon signed‐rank test for both the SMM and TPM. The remaining populations of both species presented deviations based on their *p*‐values (*p *<* *.05).

### 3.4 Global structure and migrants

The estimated *F*_IS_ suggested that *P. rubriflora* has a panmictic genic distribution (0.042) because this value did not significantly differ from zero according to the CI (Table 5). Conversely, *P. ruscifolia* had a high *F*_IS_ value (0.138) that significantly differed from zero, suggesting an intrapopulational genetic structure for this taxon according to the CI. The global *F*_ST_ presented CI values that significantly differed from zero for *P. rubriflora* and *P. ruscifolia*; this difference indicates a population structure among the areas for both species. *P. ruscifolia* had a low *F*_ST_ (0.042), reflecting a low level of structure or differentiation (*F*_ST_ < 0.05); *P. rubriflora* had a slightly higher value (0.057), reflecting a moderate population structure (*F*_ST_ = 0.05–0.15) according to the review by Balloux and Moulin (2002). The global endogamy coefficient for all populations (*F*_IT_) significantly differed from zero; this result was expected because this analysis consists of the sum of *F*_IS_ and *F*_ST_. The number of migrants (*N*_m_) could be estimated based on the *F*_ST_ values, with high values for both species (3.41 for *P. rubriflora* and 4.71 for *P. ruscifolia*). These values suggest that a large *N*_m_ helps maintain genetic homogeneity among all the investigated chaquenian areas.

**Table 5:** Global Wright’s F statistics for the 11 sampling areas of *Prosopis rubriflora* and *Prosopis ruscifolia* with their respective confidence intervals (CIs) and number of migrants *N*_m_

	*Prosopis rubriflora*				*Prosopis ruscifolia*			
	*F*_IS_	*F*_ST_	*F*_IT_	*N*_m_	*F*_IS_	*F*_ST_	*F*_IT_	*N*_m_
Estimate	0.042	0.057	0.097	3.41	0.138	0.042	0.174	4.71
Lower limit of the CI	−0.005	0.040	0.051	‐	0.053	0.034	0.093	‐
Upper limit of the CI	0.185	0.082	0.239	‐	0.226	0.050	0.257	‐

*F*_IS_, fixation index and intrapopulational endogamy; *F*_ST_, fixation index between populations; *F*_IT_, total endogamy. Confidence interval (CI) (95%): 10,000 permutations.

The AMOVA results demonstrated that most of the genetic variation (92% for *P. rubriflora* and 94% for *P. ruscifolia*) is retained within the populations sampled, with smaller portions of 8% and 6% for those two species, respectively (Table 6).

**Table 6:** Analysis of molecular variance (AMOVA) of 11 sampling areas of *Prosopis rubriflora* and *Prosopis ruscifolia*

Source of variation	*Prosopis rubriflora*			*Prosopis ruscifolia*		
	Mean square	Variance (%)	Φ ‐ *F*_ST_	Mean square	Variance (%)	Φ ‐ *F*_ST_
Between areas	14.248	7.66	0.0767	16.405	6.047	0.060
Within areas	5.065	92.337		5.860	93.953	
Total	5.448	100.000		6.203	100.000	

Simulated *p*‐value for both species *p *< .001 based on 20,000 replicates.

The Mantel test revealed a positive and significant relationship between geographic and genetic distances based on Nei’s distance (*r* = .15; *p*‐value < .01) for *P. ruscifolia* and (*r* = .2; *p*‐value < .01) *P. rubriflora*, indicating significant isolation‐by‐distance across all the sampled areas. However, considering only the areas sampled in Porto Murtinho, no such correlation was observed for *P. rubriflora* (*r* = .03; *p*‐value = .10) or *P. ruscifolia* (*r* = 0.03; *p*‐value = .09).

The pairwise *F*_ST_ ranged from −0.008 to 0.273 for *P. rubriflora* and from 0.011 to 0.101 for *P. ruscifolia* (Table S9). Based on the *F*_ST_ index discussed by Balloux and Moulin (2002), almost all Porto Murtinho areas presented low levels of structure (*F*_ST_ = 0.00–0.05), with intermediary levels between FSV and FRC1 (*F*_ST_ = 0.05–0.15) and high (*F*_ST_ = 0.15–0.25) to very high (*F*_ST_ > 0.25) levels of genetic structure (differentiation) between the areas in Porto Murtinho compared with those in the Nioaque county area (AAL).

For *P. ruscifolia*, the level of genetic structure was low for 65% of the area combinations in both sampled counties and intermediary for 35% of the combinations. An intermediary genetic structure was primarily observed for the ECD area (Corumbá); the distance between this area and the other areas in Porto Murtinho ranged from 189 to 244 km. Even with a moderate level of structure, the distinction between the sampled areas of *P. ruscifolia* was not clear (Table S9).

**Figure 2:**
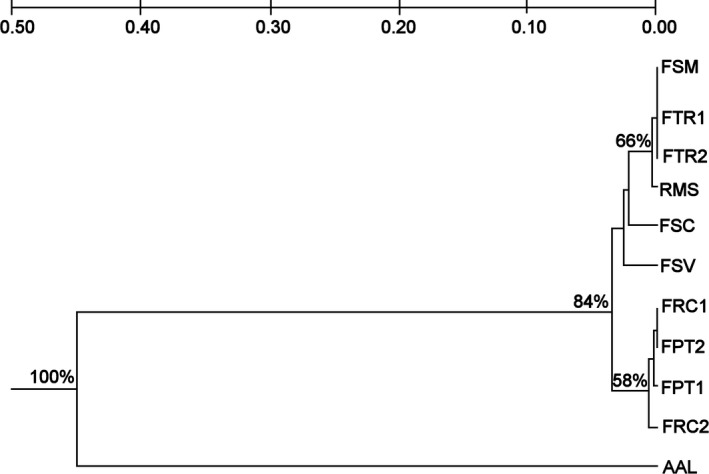
Dendrogram of the 11 sampled areas of *Prosopis rubriflora,* as determined by Nei’s genetic distance. The sampled area codes are presented in Table 1. Matrix derived from ten SSR markers, as defined by the unweighted pair‐group method of analysis (UPGMA) with 10,000 replicates

Nei’s genetic distance (Nei, 1978) varied from 0.000 to 0.450 for *P. rubriflora* (Figure 2) and was divided into two groups: the first group was in Nioaque (AAL), and the second group was composed of all areas in Porto Murtinho. The second group had two subdivisions, the areas FPT1, FPT2, FRC1, and FRC2, which are geographically and genetically closer to each other. Although Nei’s genetic distance presented these subdivisions, all the sampled areas did not appear to be substantially genetically different from each other, suggesting substantial gene flow among the areas and reinforcing the pairwise *F*_ST_ results.

**Figure 3:**
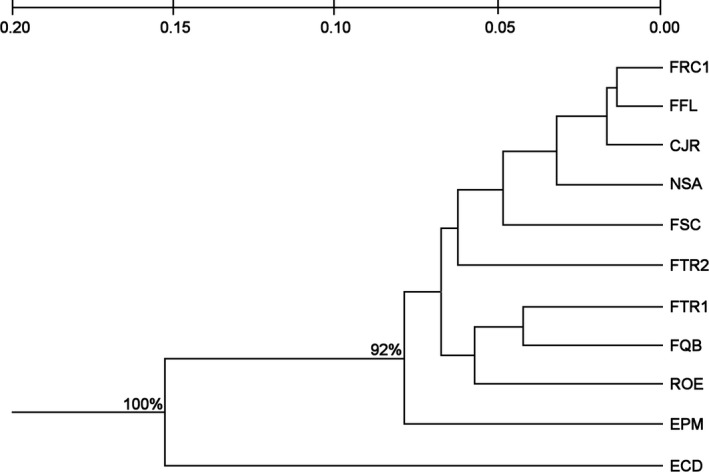
Dendrogram of the 11 sampled areas of *Prosopis ruscifolia* as determined by Nei’s genetic distance. The sampled area codes are presented in Table 1. Matrix derived from 11 SSR markers as defined by the unweighted pair‐group method of analysis (UPGMA) with 10,000 replicates

For *P. ruscifolia*, the variation in Nei’s genetic distance (Nei, 1978) between two areas ranged from 0.014 to 0.153. This species presented two major groups, ECD (Corumbá), which is the most distant area geographically, and all sampled areas in Porto Murtinho (Figure 3). The Porto Murtinho group presented two clusters: one formed with the EPM area, and the second grouped the other sampled areas supporting the *F*_ST_ pairwise results.

**Figure 4:**
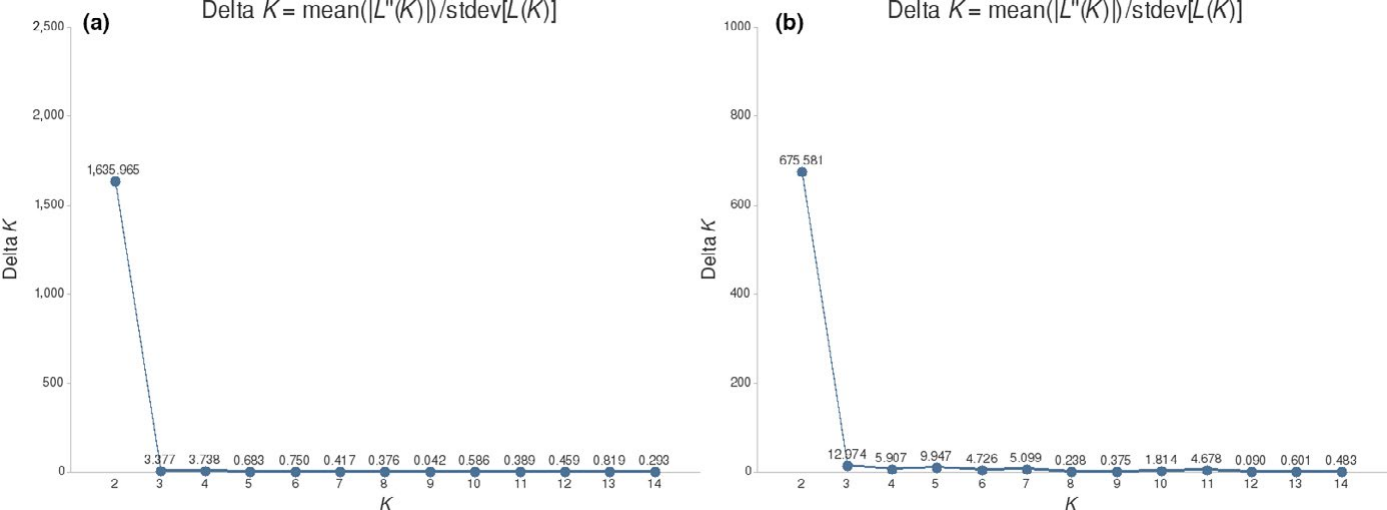
∆*K* values for all sampled areas of *Prosopis rubriflora* and *Prosopis ruscifolia*. (a) Represents *P. rubriflora*, and (b) represents *P. ruscifolia*. The values were determined based on the average of *L* (*K*) for 10 resamplings according to the model proposed by Evanno et al. (2005), and the graphics were generated by Clumpak (Kopelman et al., 2015). The *K* value represents the most likely ∆*K* according to the highest peak

**Figure 5:**
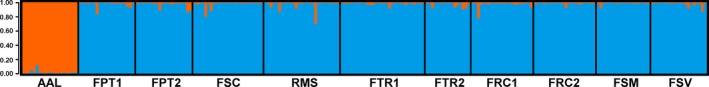
Population structure based on Bayesian analysis of the 11 stepic savanna areas sampled for *Prosopis rubriflora*. The sampled area codes are presented in Table 1. Each bar represents one sampled individual as estimated by ten SSR markers (*n *= 241)

Bayesian analyses performed with Structure software estimated the most likely number of populations (∆*K*) based on the Evanno analysis (Evanno et al., 2005). The results reported from Clumpak suggested that *K* = 2 for *P. rubriflora* (Figure 4a), which revealed two distinct clusters, a cluster in Nioaque (AAL) and another cluster in Porto Murtinho (representing all other areas) (Figure 5).

**Figure 6:**
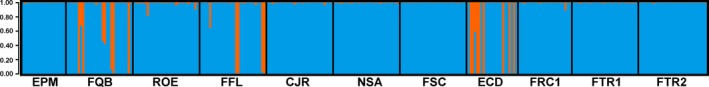
Population structure based on Bayesian analysis of the 11 stepic savanna areas sampled for *Prosopis ruscifolia*. The sampled area codes are presented in Table 1. The individuals are represented by bars (308), and the analysis is based on 11 SSR markers according to *K *= 2

In *P. ruscifolia*, the Δ*K* value obtained by the model of Evanno et al. (2005) suggested the existence of two populations (Figure 4b), but a distinct separation among these populations was not observed according to the results from Structure (Figure 6).

**Figure 7:**
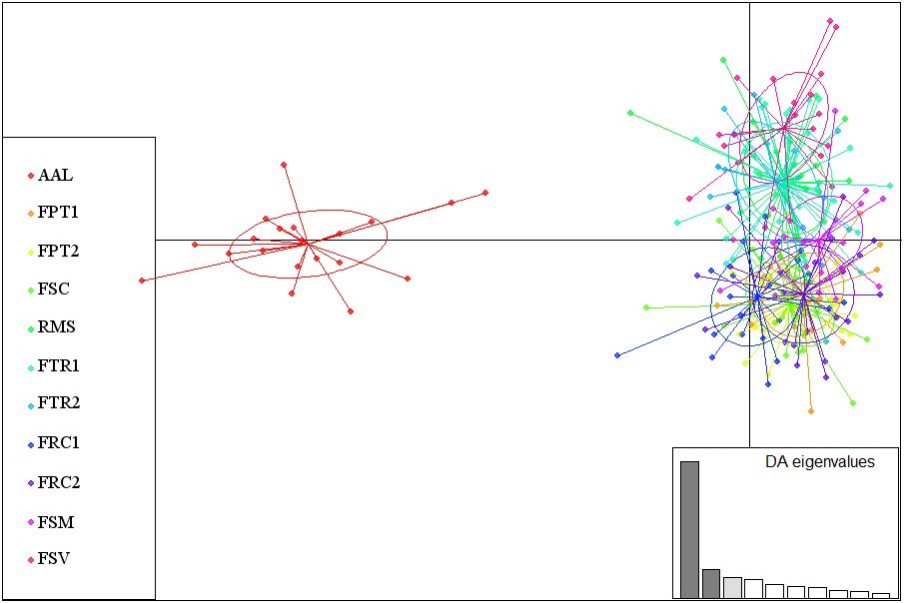
Scatterplots of DAPC using 3 discriminant functions for the 11 chaquenian areas sampled for *Prosopis rubriflora*. The sampled area codes are presented in Table 1. The plots represent the individuals, and the circles represent the groups of areas

**Figure 8:**
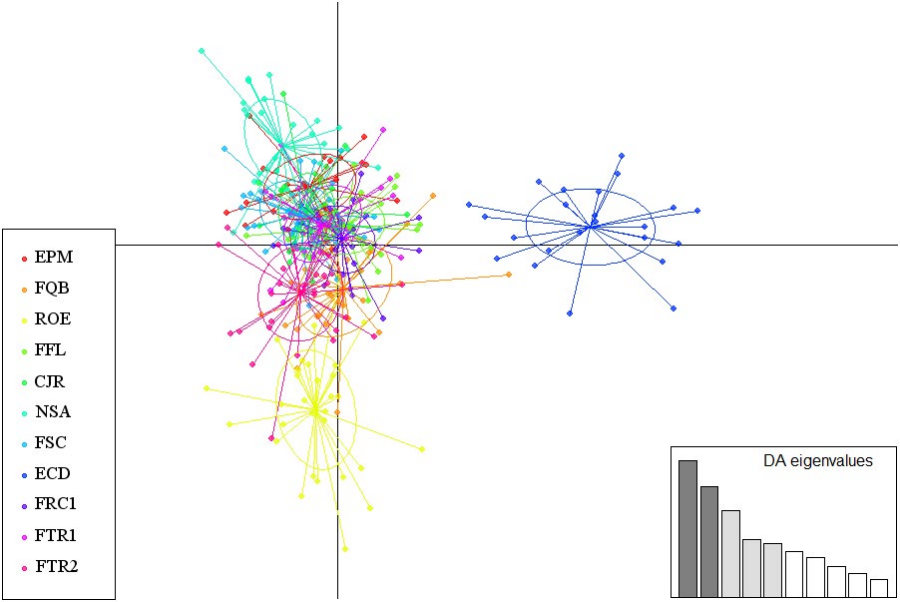
Scatterplots of DAPC using five discriminant functions for the 11 chaquenian areas sampled for *Prosopis rubriflora*. The sampled area codes are presented in Table 1. The plots represent the individuals, and the circles represent the groups of area

For the DAPC analysis, based on the number of samples obtained (241 for *P. rubriflora*), 80 axes were included, and based on the DA eigenvalues, three discriminant functions were selected, accounting for 73% of the genetic variability. For *P. ruscifolia* (308 samples obtained), 102 axes were selected, and five discriminant functions were analyzed, accounting for 71% of the genetic variability (Figures 7 and 8). The scatterplot of the individuals for both the analyzed species presented two principal components of DAPC, where one clear cluster as observed for the AAL sampling area for *P. rubriflora*, and the second cluster comprised all the other sampled areas (Figure 7). Similarly, the DAPC also presented two principal components for *P. ruscifolia*, where ECD was the only area that did not present an overlay of individuals from the other areas (Figure 8).

## 4 DISCUSSION

Small and isolated populations may undergo biparental inbreeding, which causes a loss of genetic diversity and inbreeding depression and may reduce the ability of the population to respond to environmental changes (Frankham et al., 2008). Thus, genetic diversity within populations is fundamental for conservation biology; better adaptation potential is expected in populations with high levels of genetic diversity (Kalinowski, 2004). The main parameters that are used to evaluate the intrapopulational genetic diversity include allelic diversity, *H*_o_, *H*_e*,*_ and the inbreeding coefficient (Berg & Hamrick, 1997).

A tendency toward the homogeneity of alleles among the areas sampled in Porto Murtinho was observed when considering the *A*_ri_ of *P. rubriflora*. This pattern may reflect fewer disturbed areas or areas that have not yet experienced sufficiently strong suppression to display more sudden fluctuations. The areas that exhibited significant *A*_ri_ compared with the other areas, such as FSV and RMS, may receive major influxes of migrants compared with other areas. This influence may be reflected in the *A*_ri_, which corrects for differences among samples for all areas (El Mousadik & Petit, 1996). In the same county, the FTR2 area showed high numbers of effective alleles; this possibly reflects the suppression observed in the area and a loss of individuals with *A*_r_. Similar results for disturbed areas were also observed for *P. reticulata* Benth. (Oliveira, 2012) and *S. lycocarpum* (Moura, 2007).

The lowest allelic diversity was recorded in the disjunct area of Nioaque (AAL); however, this area lacks direct evidence of anthropic disturbance. This population may have been small for a long period and must have experienced both loss and genetic reorganization, which was expected due to genetic drift (Ellstrand & Elam, 1993). The factors responsible for the low diversity should be related to the geographic distance and the lack of connection between this area and other areas in Porto Murtinho (Figure 1).

For *P. ruscifolia*, the diversity and richness of alleles were heterogeneous in configuration due to the different degrees of disturbance in the sampling sites or the reproductive system of the species. The area with the greatest diversity of alleles was FFL, which had less apparent disturbance. Although the diversity and *A*_ri_ in the FQB and ECD areas were similar to the diversity and *A*_ri_ in the FFL areas, these regions had the highest levels of disturbance of all the sampled areas. Similar results for disturbed areas were recorded by Barreto in *D. nigra* (Barreto, 2010). The ECD and FQB areas may have also undergone recent suppression, in which most of the *P. ruscifolia* individuals were preserved; this preservation was unlike that of all other tree species and hindered the detection of any allelic loss. Adult *P. ruscifolia* are frequently observed in highly disturbed areas, such as pastures, where these trees may not be cut down for practical reasons (to provide shade for livestock), esthetic reasons, or difficulties in cutting.

The genetic diversity values were similar in *P. rubriflora* and *P. ruscifolia*; this result was not expected because *P. rubriflora* had reduced geographic distributions in Brazil and Paraguay compared with *P. ruscifolia,* which also occurred in Argentina and Bolivia (Burkart, 1976). Our initial hypothesis was that *P. ruscifolia* would show higher genetic diversity, as noted by Hamrick and Godt (1996) and Nybom (2004). Assuming the older and current anthropogenic disturbances were not sufficiently strong to cause similar *H*_e_ values for both species, the possibility that the combination of biological factors, such as the different flowering periods for these species, were restricted for *P. ruscifolia* and extended for *P. rubriflora* could explain the results obtained herein.

The difference between the *H*_e_ and *H*_o_ parameters was lower in *P. rubriflora* than in *P. ruscifolia* for all areas sampled in Porto Murtinho and resulted in lower deviations from HW proportions and a lower inbreeding coefficient. The inbreeding coefficient significantly differed from zero in *P. rubriflora* only for the area in Nioaque; the AAL area revealed little disturbance. Because AAL contains a small population and is geographically isolated, stronger evidence of inbreeding was expected, especially in the biparental data and in terms of its structure due to limitations in pollen flow and seed dispersal (Ellstrand & Elam, 1993).

Both AAL and ECD were the only areas outside of Porto Murtinho county where we were able to find *P. rubriflora* and *P. ruscifolia*, respectively, in Brazilian chaquenian areas. The disjunctive area of Nioaque, close to Morro do Solteiro, was the only area registered for this county. For Corumbá County, there were a few areas wherein *P. ruscifolia* was registered in previous years; however, we were unable to find these areas for this study, most likely because they were suppressed months before our visit. Other areas with *P. ruscifolia* possibly exist south of Corumbá, but as the access for this region is very difficult, we were unable to seek additional areas for this county.

For conservation measures, AMOVA suggests that most genetic variations are retained inside the populations, indicating the importance of protecting the sampled areas to avoid losses in genetic variability for both species. Even though all the sampled areas contain a precious genetic resource, conservation management of the AAL should be more prioritized in the short term due to its small population size and reduced genetic diversity compared with those of the Porto Murtinho areas. This action is necessary to conserve the current genetic diversity and avoid further reductions in responsiveness to environmental changes, which increase the likelihood of local extinction (Frankham et al., 2008) and may be permanent.

In *P. ruscifolia,* the major differences between *H*_e_ and *H*_o_ were reflected in larger deviations from HW proportions and, consequently, higher values of *F*_IS_; these differences suggest endogamy and an intrapopulational structure for 64% of the sampled areas. The isolated population of *P. rubriflora* in Nioaque had the highest *F*_IS_, whereas the distant Corumbá area (ECD) had the highest level of intrapopulational structure for *P. ruscifolia*. In addition, the geographic isolation of the severely disturbed ECD area may contribute to intrapopulational inbreeding to maximize the genetic structure. Similar levels of inbreeding were detected by Bessega et al. (2000) in *P. ruscifolia*; these authors reported values of *T*_*m*_ that were similar to the average *t*_*a*_ obtained in this study for the same species, thereby supporting the results obtained by the apparent crossing rate in this study.

Based on the *t*_a_ index results, *Eugenia dysenterica* DC (Zucchi et al., 2003) and *Solanum lycocarpum* A. St‐Hil. (Moura, 2007) presented low inbreeding, similar to *P. rubriflora*, even in disturbed or regeneration areas. Wild plants that are less endogamic are expected to respond better to deleterious effects, such as bottlenecks; therefore, the effect of inbreeding depression on these populations should be less pronounced in future generations. Conversely, the remaining species, including *P. ruscifolia*, may present a mixed mating system and be less limiting for inbreeding. Consequently, reductions in the allelic frequency and in heterozygotes are expected and possibly render these species more sensitive to environmental disturbances over time.

The taxa *P. rubriflora* and *P. ruscifolia* present higher values of genetic diversity compared with those of *P. alba* and *P. ruscifolia* sampled from Santiago del Estero, Argentina, according to the findings of Ferreyra, Vilardi, Verga, López, & Saidman (2013); however, this difference can be attributed to the use of a different molecular marker and the lower number of samples used by Ferreyra et al. *P. rubriflora* and *P. alba* have average values of *H*_e_ compared with those obtained in other studies using SSR markers on South American arboreal taxa, such as *C. langsdorffii* (Martins, Santos, Gaiotto, Moreno, & Kageyama, 2008), *D. nigra* (Barreto, 2010), *D. alata* (Collevatti et al., 2013), *E. dysenterica* (Zucchi et al., 2003), *L. divaricata* (Conson et al., 2013), *P. reticulata* (Oliveira, 2012), *Q. grandiflora* (Ritter, 2012), and *S. lycocarpum* (Moura, 2007). However, the *F*_IS_ from these studies suggests that *Prosopis* populations may experience smaller effects of environmental disturbances than populations of *D. nigra* (Barreto, 2010), *P. reticulata* (Oliveira, 2012), *Q. grandiflora* (Ritter, 2012), *C. langsdorffii* (Martins et al., 2008), *D. alata* (Collevatti et al., 2013), and *L. divaricata* (Conson et al., 2013). The Pantanal has a recent history of degradation that is signified by the loss of native flora to extensive livestock agriculture; this livestock agriculture was implemented aggressively beginning in 1976 (Abdon et al., 2007). Thus, estimating the degree of loss in genetic variability in the chaquenian areas of the South Pantanal region may not be feasible at this time.

However, Porto Murtinho was an important component of the Yerba Mate cycle from the late nineteenth century until the middle of the twentieth century (Silva, 1997). Suppression of the native forest in the Chaco to enable the planting of *Ilex paraguariensis* A. St.‐Hil. or for other economic benefits could be responsible for the observed deviations in *H*_e_ and *H*_o_ and may be reflected in high levels of fixation in many of the sampled areas for *P. ruscifolia*.

*N*_e_ estimates how many individuals will genetically contribute to the next generation (Nunney & Campbell, 1993), and the estimated values of *N*_e_/*N* were 0.25–1.00 (Ellstrand & Elam, 1993; Nunney & Campbell, 1993). Averages of 43% of trees (in *P. ruscifolia*), ranging from 31% in ECD to 49% in FSC, and 54% of trees (in *P. rubriflora*), ranging from 45% (AAL) to 59% (FPT1 and FSM), will genetically contribute to the Chaco areas, and the largest oscillations have generally occurred in disturbed areas or smaller populations. According to Frankham et al. (2008), an estimated *N*_e_ of 12–1,000 is required to prevent the accumulation of deleterious mutations, whereas an *N*_e_ of 50 is sufficient to avoid inbreeding depression, and an *N*_e_ of 500 is sufficient to retain the evolutionary potential over 100–1,000 generations. Thus, the areas ECD and AAL (a disjunct area with unknown connectivity) may be at risk for accumulating deleterious mutations due to their isolation. Although the Porto Murtinho areas have reduced effective populations, they have greater connectivity and should not be at risk for inbreeding depression; however, because the total *N*_e_ is <500, a minimum of 42 Chaco areas of similar size should be preserved to maintain the evolutionary potential of both species.

From all 18 different sampled areas, AAL, FPT2, FTR2, FRC2, EPM, FQB, ROE, CJR, FSC, and ECD did not appear to have experienced a bottleneck effect. These deviations suggest that 64% of the *P. rubriflora* and 36% of the *P. ruscifolia* remnants have undergone bottlenecks; however, the excessive H.d. observed from the sign test of most remnant areas suggests a population expansion or the introduction of *A*_r_ from immigrants (Luikart & Cornuet, 1998). The bottleneck effect can be attributed to natural mortality (diseases and climate changes) and artificial (anthropic) causes; thus, previous knowledge is required to define the effect of the bottleneck on the species. As such, the areas under bottleneck in this study could be due to older natural disturbances, such as flooding and storms, or artificial disturbances, such as anthropogeny for older economic activities. However, except the areas FPT2 and FRC2, most of the sampled areas free from the bottleneck effect are currently under anthropogenic disturbances to higher or lower degrees, mainly for cattle breeding according to our field observations, which is expected to result in the bottleneck effect for the next generations in most chaquenian areas.

The population structure observed among the populations of *P. rubriflora* considered the deviation that was observed in the CI_95%_. The low intrapopulational structure of *P. rubriflora* may be related to flowering throughout the year and to reproductive systems that are less tolerant to selfing. On the other hand, *P. ruscifolia* showed structure both within and among populations according to the global analysis even with a lower *F*_ST_ value of 0.05, suggesting a significant but weak interpopulational structure for *P. ruscifolia* (Balloux & Moulin, 2002).

Refined estimates of genetic structure using pairwise Mantel test, *F*_ST_, Nei’s genetic distance, DAPC, and Bayesian structural inferences provided additional evidence of low levels of structure in nearly all the Porto Murtinho areas, suggesting a high gene flow in the investigated species for this county. The dendrogram Nei’s distance, which yields a refined analysis based on the genetic similarity of the sampled populations, presented few branches with well‐supported nodes (<50%). This result reinforces the high gene flux for most of the sampled Porto Murtinho areas, making determining differences from each other difficult. DAPC revealed a very strong relationships among all the sampled areas in Porto Murtinho for both *P. rubriflora* and *P. ruscifolia*, supporting the Bayesian analysis in Structure and consistent across the genetic structure analyses. As such, the low genetic structure among the Porto Murtinho areas regardless of geographic distance could be due to the connections between the areas, which enable a longer gene flow for both species. Migrants may also contribute to the genetic homogeneity in these areas (Varvio, Chakraborty, & Nei, 1986).

Compared with all the Porto Murtinho areas in all the analyses, the taxon *P. rubriflora* showed a distinct genetic structure in AAL (Nioaque), supported by all the analyses. This structure is likely the result of geographic distance (ca. 218–267 km) and the lack of connection between these areas; this lack of connection severely limits pollen flow and causes long periods of isolation for AAL. This disjunction appears to have resulted from ancient natural factors rather than from recent anthropogenic fragmentation. *Prosopis* grows poorly in acidic and low‐phosphorus soils (Pasiecznik et al., 2001), such as soils in the Cerrado (Oliveira, Costa, Santos, & Moreira, 2005; Sano, 1998) between Porto Murtinho and Nioaque; this growth property limits the distribution of this genus in Brazil and in another regions wherein the genus occurs natively or was introduced (Figure 1).

The genetic structure of *P. ruscifolia* showed low‐to‐moderate variations in the pairwise *F*_ST_ analysis; in this analysis, a strong structure was observed between the remaining area of Corumbá (ECD) and other Porto Murtinho areas. Although the distance between the ECD area (Corumbá) and the remainder of Porto Murtinho (ca. 189–244 km) was similar to that affecting *P. rubriflora*, the moderate structure indicates that these regions can be connected, assuring some level of gene flow. However, the continuous fragmentation of chaquenian areas used for cattle farming was expected to increase the structure in this area compared with the remainder of Porto Murtinho. In addition to the observed moderate population structure of *P. ruscifolia* in the ECD area, a similar structure was estimated between some Porto Murtinho areas by pairwise analysis. These lower‐level yet significant structures may be attributed to the fragmentation of forested areas and the shorter flowering and fruiting period, which, in turn, may contribute to the lower gene flow compared with that of *P. rubriflora*.

Thus, *P. rubriflora* has two distinct populations in the areas sampled in Mato Grosso do Sul that are produced by an ancient isolation event. Although *P. ruscifolia* apparently has some connections with the other areas according to the *F*_ST_ pairwise, Nei’s genetic distance, and Structure analyses, this connection appears limited according to the DAPC and Mantel test results. If the anthropogenic disturbance and fragmentation remain constant, the area in Corumbá will become increasingly isolated and possibly present results similar to those observed in the Nioaque area for *P. rubriflora*, which has reduced genetic variability and a significant population structure.

We expect the results of this study to help with the allotment of subsidies for decision making and the development of conservation strategies for chaquenian areas. These measures will help to preserve the genetic stock for both *Prosopis* species and other rare species that occur only in this biome, which is increasingly suppressed by anthropic pressures.

## 5 CONCLUSIONS

The genetic diversity (*H*_e_) of *P. rubriflora* appears to be similar to that of *P. ruscifolia*. Although *P. ruscifolia* has more alleles, most of the alleles are rare and do not increase *H*_e_ to the same degree. The apparent conservation status of an area can be misleading regarding allelic diversity, and even disturbed areas may have high allelic diversity. Evidence of a bottleneck was detected for both species, and *P. rubriflora* was affected in most of the analyzed areas.

High gene flow was observed between populations, and a strong structure was evidenced only in extreme cases, such as populations at a substantial geographic distance and with a lack of connection. The intrapopulation structure was higher for *P. ruscifolia*, as expected. Despite the predominance of bees as pollen dispersal agents, a relatively small structure index was observed between the sampled areas; this small index indicates high gene flow because the connection between areas enables pollen flow. Based on the effective population number in this study, 42 Chaco areas must be preserved to preserve the minimum of 500 individuals needed to maintain genetic diversity and retain the evolutionary potential of both species over 100–1,000 generations. The measures suggested in this study should prevent additional environmental damage that may cause extinction, which would negatively affect the local fauna because *P. rubriflora* and *P. ruscifolia* provide important food resources.

## CONFLICT OF INTEREST

None declared.

## AUTHOR CONTRIBUTION

Alves FM, Zucchi MI, and Souza AP involved in conception and designed the work; Alves FM and Sartori ALB acquired the data; Alves FM, Zucchi MI, Souza AP, Tambarussi EV, and Alves‐Pereira A developed the analysis and interpreted the results; Alves FM, Zucchi MI, Souza AP, Tambarussi EV, Sartori ALB, Azevedo‐Tozzi AMG, and Alves‐Pereira A drafted the article and reviewed the content; Alves FM, Zucchi MI, Souza AP, Tambarussi EV, Sartori ALB, Azevedo‐Tozzi AMG, and Alves‐Pereira A made final approval of the version to be published.
