# Supplementary material for: Genetic structure of two Prosopis species in Chaco areas: A lack of allelic diversity diagnosis and insights into the allelic conservation of the affected species
Source: Ecol Evol. 2018 Jun 7;8(13):6558–74. doi: 10.1002/ece3.4137 (PMC6053563; doi:10.1002/ece3.4137)
Supplement: Supplementary file 1 [file ECE3-8-6558-s001.docx]

**Supporting Information**

**Table S1. Test of adherence to Hardy-Weinberg equilibrium according to Fisher’s exact probability performed on areas sampled for *Prosopis rubriflora*.**

| **Marker** | ***P-*value** | | | | | | | | | | |
| --- | --- | --- | --- | --- | --- | --- | --- | --- | --- | --- | --- |
|  | **AAL** | **FPT1** | **FPT2** | **FSC** | **RMS** | **FTR1** | **FTR2** | **FRC1** | **FRC2** | **FSM** | **FSV** |
| **Prb1** | 0.132  (0.001) | 0.636  (0.003) | 0.043  (0.001) | 0.498  (0.003) | 0.044  (0.002) | 0.453  (0.005) | 0.793  (0.001) | 0.095  (0.002) | 0.184  (0.002) | 0.838  (0.002) | 0.2631  (0.004) |
| **Prb2** | **0.003**  (0.001) | 1.000  (0.000) | 1.000  (0.000) | 1.000  (0.000) | 1.000  (0.000) | 1.000  (0.000) | 0.097  (0.001) | 1.000  (0.000) | 0.139  (0.002) | - | 1.000  (0.000) |
| **Prb3** | - | 1.000  (0.000) | 1.000  (0.000) | 1.000  (0.000) | 0.615  (0.001) | 1.000  (0.000) | 1.000  (0.000) | 1.000  (0.000) | - | 0.026  (0.001) | 0.031  (0.001) |
| **Prb4** | 0.360  (0.007) | 0.373  (0.011) | 0.521  (0.010) | 0.330  (0.017) | 0.039  (0.007) | **0.001**  (0.001) | **0.000**  (0.000) | 0.013  (0.004) | 0.108  (0.006) | 0.532  (0.013) | 0.238  (0.008) |
| **Prb5** | 0.951  (0.001) | 0.006  (0.001) | 0.051  (0.001) | 0.112  (0.002) | 0.492  (0.006) | 0.211  (0.004) | 0.316  (0.002) | 0.167  (0.003) | 0.975  (0.001) | 1.000  (0.000) | 0.111  (0.001) |
| **Prb6** | 0.460  (0.004) | 0.506  (0.005) | 0.066  (0.003) | 0.378  (0.005) | 0.136  (0.004) | 0.663  (0.003) | 0.151  (0.005) | 0.639  (0.003) | 0.006  (0.001) | 0.800  (0.002) | 0.270  (0.009) |
| **Prb7** | 1.000  (0.000) | 0.077  (0.001) | 1.000  (0.000) | 0.078  (0.001) | 0.848  (0.001) | 0.174  (0.002) | 0.207  (0.001) | 1.000  (0.000) | 0.110  (0.001) | 0.760  (0.001) | 0.604  (0.002) |
| **Prb9** | 0.142  (0.004) | 0.031  (0.002) | 0.206  (0.008) | 0.462  (0.010) | 0.787  (0.004) | 0.572  (0.010) | 0.942  (0.003) | 0.664  (0.007) | 0.114  (0.004) | 0.494  (0.010) | 0.630  (0.006) |
| **Prs1** | 0.050  (0.002) | 0.344  (0.004) | 0.185  (0.004) | 0.648  (0.005) | 0.566  (0.006) | 0.750  (0.006) | 0.062  (0.002) | 0.518  (0.005) | 0.122  (0.006) | 0.571  (0.003) | 0.831  (0.006) |
| **Prs11** | 0.070  (0.001) | 0.030  (0.002) | 0.834  (0.003) | 0.274  (0.007) | 0.723  (0.009) | 0.509  (0.009) | 0.091  (0.003) | 0.236  (0.004) | 0.235  (0.004) | 0.166  (0.006) | 0.596  (0.004) |

The sampled area codes are presented in Table 1. The test was developed with 10,000 randomizations. The bold values represent the loci departed from Hardy-Weinberg equilibrium after Bonferroni correction (*P* < 0.005); the standard errors (SEs) are indicated in parentheses.

**Table S2. Analysis to detect null alleles for SSR markers evaluated for the sampled *Prosopis rubriflora* populations.**

| **Marker** | **Null alleles** | | | | | | | | | | |
| --- | --- | --- | --- | --- | --- | --- | --- | --- | --- | --- | --- |
|  | **AAL** | **FPT1** | **FPT2** | **FSC** | **RMS** | **FTR1** | **FTR2** | **FRC1** | **FRC2** | **FSM** | **FSV** |
| **Prb1** | 0.082 | 0.039 | 0.072 | 0.057 | 0.043 | 0.068 | 0.048 | 0.073 | 0.054 | 0.000 | 0.022 |
| **Prb2** | **0.221** | 0.000 | 0.000 | 0.000 | 0.000 | 0.000 | 0.138 | 0.000 | 0.110 | 0.001 | 0.000 |
| **Prb3** | - | 0.000 | 0.000 | 0.000 | 0.029 | 0.000 | 0.000 | 0.000 | - | 0.123 | 0.157 |
| **Prb4** | 0.035 | 0.050 | 0.041 | 0.041 | 0.019 | 0.052 | 0.187 | 0.047 | 0.032 | 0.020 | 0.000 |
| **Prb5** | 0.000 | 0.011 | 0.000 | 0.000 | 0.000 | 0.000 | 0.057 | 0.083 | 0.000 | 0.014 | 0.042 |
| **Prb6** | 0.000 | 0.000 | 0.082 | 0.000 | 0.046 | 0.000 | 0.026 | 0.043 | 0.119 | 0.000 | 0.000 |
| **Prb7** | 0.020 | 0.000 | 0.028 | 0.000 | 0.000 | 0.000 | 0.000 | 0.000 | 0.000 | 0.000 | 0.000 |
| **Prb9** | 0.000 | 0.038 | 0.062 | 0.000 | 0.000 | 0.000 | 0.000 | 0.000 | 0.015 | 0.022 | 0.000 |
| **Prs1** | 0.153 | 0.023 | 0.000 | 0.017 | 0.000 | 0.000 | 0.000 | 0.015 | 0.015 | 0.000 | 0.000 |
| **Prs11** | 0.000 | 0.060 | 0.000 | 0.000 | 0.000 | 0.000 | 0.000 | 0.000 | 0.031 | 0.046 | 0.000 |
| **Average** | 0.06 | 0.02 | 0.03 | 0.01 | 0.01 | 0.01 | 0.05 | 0.03 | 0.04 | 0.02 | 0.02 |

The sampled area codes are presented in Table 1. The bold values represent the loci with possible null alleles for each sampled area in which the null frequency was < 0.20.

**Table S3. Pairwise analysis of linkage disequilibrium in ten SSR loci in *Prosopis rubriflora.***

| **Loci pairwise** | ***P-*Value** | **Loci pairwise** | ***P-*Value** |
| --- | --- | --- | --- |
| Prb6_&_Prb1 | 0.7292 | Prb3_&_Prb9 | 0.6571 |
| Prb6_&_Prb2 | 0.6728 | Prs1_&_Prb9 | 0.6557 |
| Prb1_&_Prb2 | 0.2761 | Prb6_&_Prb4 | 0.9943 |
| Prb6_&_Prb5 | 0.6491 | Prb1_&_Prb4 | 0.3776 |
| Prb1_&_Prb5 | 0.5646 | Prb2_&_Prb4 | 0.3538 |
| Prb2_&_Prb5 | 0.6340 | Prb5_&_Prb4 | 0.1439 |
| Prb6_&_Prb7 | 0.2997 | Prb7_&_Prb4 | 0.5727 |
| Prb1_&_Prb7 | 0.1287 | Prb3_&_Prb4 | 0.8062 |
| Prb2_&_Prb7 | 0.3712 | Prs1_&_Prb4 | 0.9967 |
| Prb5_&_Prb7 | 0.9415 | Prb9_&_Prb4 | 0.9109 |
| Prb6_&_Prb3 | 0.8551 | Prb6_&_Prs11 | 0.9488 |
| Prb1_&_Prb3 | 0.1266 | Prb1_&_Prs11 | 0.2439 |
| Prb2_&_Prb3 | 0.9166 | Prb2_&_Prs11 | 0.9878 |
| Prb5_&_Prb3 | 0.2448 | Prb5_&_Prs11 | 0.6264 |
| Prb7_&_Prb3 | 0.0275 | Prb7_&_Prs11 | 0.1422 |
| Prb6_&_Prs1 | 0.4317 | Prb3_&_Prs11 | 0.9567 |
| Prb1_&_Prs1 | 0.9732 | Prs1_&_Prs11 | 0.7833 |
| Prb2_&_Prs1 | 0.7979 | Prb9_&_Prs11 | 0.7211 |
| Prb5_&_Prs1 | 0.0939 | Prb4_&_Prs11 | 0.9969 |
| Prb7_&_Prs1 | 0.9852 | - | - |
| Prb3_&_Prs1 | 0.6062 | - | - |
| Prb6_&_Prb9 | 0.9967 | - | - |
| Prb1_&_Prb9 | 0.8033 | - | - |
| Prb2_&_Prb9 | 0.1112 | - | - |
| Prb5_&_Prb9 | 0.3401 | - | - |
| Prb7_&_Prb9 | 0.9768 | - | - |

All areas were evaluated according to Fisher's exact probability test. Linkage disequilibrium was considered absent at all the analysed loci for all *P. rubriflora* sampling areas with *P*-values at 1% (0.0002) and 5% (0.0011) after Bonferroni correction.

**Table S4. Fisher's exact test estimates of *F*_IS_ in the sampled areas for both studied species.**

| *Prosopis rubriflora* |  | **AAL** | **FPT1** | **FPT2** | **FSC** | **RMS** | **FTR1** | **FRT2** | **FRC1** | **FRC2** | **FSM** | **FSV** |
| --- | --- | --- | --- | --- | --- | --- | --- | --- | --- | --- | --- | --- |
|  | **Prb6** | 0.4145 | 0.3687 | 0.0799 | 0.9556 | 0.4167 | 0.4289 | 0.1885 | 0.396 | 0.0149 | 0.8012 | 0.3917 |
|  | **Prb1** | 0.1859 | 0.4534 | 0.0966 | 0.182 | 0.4223 | 0.0831 | 0.2965 | 0.0823 | 0.1254 | 0.8715 | 0.2023 |
|  | **Prb2** | 0.0029 | 1.0000 | 1.0000 | 1.0000 | 1.0000 | 1.0000 | 0.0965 | 1.0000 | 0.0704 | 1.0000 | 1.0000 |
|  | **Prb5** | 0.7176 | 0.6204 | 0.9499 | 0.9914 | 0.2807 | 0.5346 | 0.3132 | 0.0969 | 0.7196 | 0.417 | 0.1757 |
|  | **Prb7** | 0.5806 | 0.9948 | 0.5176 | 0.9872 | 0.6183 | 0.9862 | 0.9484 | 0.7585 | 0.9601 | 0.6146 | 0.548 |
|  | **Prb3** | NA | 1.0000 | 1.0000 | 0.7527 | 0.4648 | 1.0000 | 1.0000 | 1.0000 | NA | 0.0961 | 0.03 |
|  | **Prs1** | 0.0114 | 0.2529 | 0.4853 | 0.5926 | 0.2467 | 0.9023 | 0.7433 | 0.1448 | 0.1198 | 0.5769 | 0.6484 |
|  | **Prb9** | 0.3071 | 0.0841 | 0.1355 | 0.9500 | 0.6153 | 0.9897 | 0.9579 | 0.7276 | 0.2603 | 0.1294 | 0.4241 |
|  | **Prb4** | 0.1228 | 0.1054 | 0.5577 | 0.246 | 0.0167 | 0.0217 | 0.0004 | 0.0567 | 0.2721 | 0.2522 | 0.9318 |
|  | **Prs11** | 0.3939 | 0.0620 | 0.8524 | 0.9334 | 0.623 | 0.6292 | 0.938 | 0.9288 | 0.1248 | 0.1673 | 0.6699 |
|  | **All** | **0.0003** | 0.1133 | 0.1825 | 0.9907 | 0.0687 | 0.7178 | 0.1189 | 0.0567 | 0.0123 | 0.0846 | 0.1064 |
| *Prosopis ruscifolia* |  | **EPM** | **FQB** | **ROE** | **FFL** | **CJC** | **NSA** | **FSC** | **ECD** | **FRC1** | **FTR1** | **FTR2** |
|  | **Prs6** | 0.0640 | 0.0000 | 0.0000 | 0.0008 | 0.0002 | 0.1962 | 0.0179 | 0.0210 | 0.0007 | 0.0000 | 0.0000 |
|  | **Prs1** | 0.4682 | 0.1356 | 0.7973 | 0.3522 | 0.8149 | 0.3357 | 0.6554 | 0.0137 | 0.7146 | 0.9523 | 0.9112 |
|  | **Prb2** | 0.2629 | 0.6949 | 0.8284 | 0.9611 | 0.3584 | 0.2093 | 0.5478 | 10.000 | 0.9243 | 0.6252 | 0.6710 |
|  | **Prs2** | 0.7135 | 0.0316 | 0.2359 | 0.0042 | 0.1319 | 0.1672 | 0.2001 | 0.0246 | 0.2157 | 0.0168 | 0.3245 |
|  | **Prb4** | 0.6159 | 0.7603 | 0.1443 | 0.0082 | 0.7692 | 0.7297 | 0.5694 | 0.3377 | 0.5413 | 0.2471 | 0.7205 |
|  | **Prs7** | 0.1614 | 0.0000 | 0.0049 | 0.0466 | 0.0001 | 0.0001 | 0.022 | 0.0021 | 0.0003 | 0.0108 | 0.0015 |
|  | **Prs11** | 0.0000 | 0.0000 | 0.8287 | 0.0000 | 0.0003 | 0.4474 | 0.2254 | 0.0000 | 0.0000 | 0.0207 | 0.0281 |
|  | **Prs12** | 0.3545 | 0.0000 | 0.3690 | 0.3106 | 0.1137 | 0.9941 | 0.9152 | 0.0232 | 0.8351 | 0.2557 | 0.6631 |
|  | **Prs3** | 0.6858 | 1.0000 | 1.0000 | 0.6110 | 0.1553 | 1.0000 | 0.7479 | 0.0711 | 0.2889 | 1.0000 | 1.0000 |
|  | **Prs5** | 0.0478 | 0.0305 | 0.9382 | 0.3153 | 0.672 | 0.9195 | 0.9885 | 0.0007 | 1.0000 | 0.257 | 0.5007 |
|  | **Prb7** | 1.0000 | 0.8641 | 1.0000 | 0.0499 | 0.2232 | 1.0000 | 1.0000 | 0.0061 | 0.0408 | 1.0000 | 1.0000 |
|  | **All** | **0.0000** | **0.0000** | 0.1080 | **0.0000** | **0.0000** | 0.0827 | 0.1887 | **0.0000** | **0.0000** | **0.0000** | 0.0029 |

The sampled area codes are presented in Table 1. The results are based on 110,000 randomizations for *P. rubriflora* and 121,000 randomizations for *P. ruscifolia***.** Adjusted nominal value after Bonferroni correction (5%); *P* = 0.00045 for *P. rubriflora* and *P* = 0.00041 for *P. ruscifolia*. Bold type indicate f values that are significantly higher than zero.

**Table S5. Test of adherence to Hardy-Weinberg equilibrium according to Fisher’s exact probability performed on areas sampled for *Prosopis ruscifolia*.**

| **Marker** | ***P-*value** | | | | | | | | | | |
| --- | --- | --- | --- | --- | --- | --- | --- | --- | --- | --- | --- |
|  | **EPM** | **FQB** | **ROE** | **FFL** | **CJC** | **NSA** | **FSC** | **ECD** | **FRC1** | **FTR1** | **FTR2** |
| **Prs6** | 0.063 (0.003) | **0.000**  (0.000) | **0.002**  (0.000) | **0.000**  (0.000) | **0.000**  (0.000) | 0.126  (0.007) | 0.077  (0.003) | **0.003**  (0.001) | **0.000**  (0.000) | **0.000**  **(**0.000) | **0.000**  (0.000) |
| **Prs1** | 0.788  (0.002) | 0.005  (0.001) | 0.857  (0.003) | 0.011  (0.002) | 0.027  (0.001) | 0.791  (0.003) | 1.000  (0.000) | **0.003**  (0.000) | 0.914  (0.002) | 0.591  (0.006) | 0.422  (0.003) |
| **Prb2** | 0.514  (0.003) | 1.000  (0.000) | 0.723  (0.002) | 0.662  (0.003) | 0.877  (0.001) | 0.575  (0.003) | 0.351  (0.004) | 1.000  (0.000) | 0.776  (0.003) | 0.506  (0.003) | 0.958  (0.001) |
| **Prs2** | 0.699  (0.007) | 0.059  (0.003) | 0.115  (0.005) | 0.008  (0.002) | 0.214  (0.011) | 0.034  (0.004) | 0.041  (0.003) | **0.001**  (0.000) | 0.033  (0.004) | 0.008  (0.001) | 0.581  (0.005) |
| **Prb4** | 0.890  (0.004) | 0.021  (0.004) | 0.355  (0.013) | 0.335  (0.010) | 0.568  (0.014) | 0.339  (0.008) | 0.751  (0.014) | 0.781  (0.017) | 0.014  (0.002) | 0.480  (0.013) | 0.708  (0.013) |
| **Prs7** | 0.175  (0.004) | **0.000**  (0.000) | 0.005  (0.001) | 0.072  (0.005) | **0.001**  (0.000) | **0.000**  (0.000) | 0.094  (0.004) | **0.000**  (0.000) | **0.000**  (0.000) | 0.005  (0.000) | 0.018  (0.002) |
| **Prs11** | **0.000**  (0.000) | **0.000**  (0.000) | 0.705  (0.016) | **0.000**  (0.000) | **0.000**  (0.000) | **0.000**  (0.000) | 0.508  (0.012) | **0.000**  (0.000) | **0.000**  (0.000) | 0.056  (0.005) | 0.323  (0.006) |
| **Prs12** | 0.138  (0.002) | **0.000**  (0.000) | 0.675  (0.005) | 0.268  (0.005) | **0.001**  (0.000) | 0.132  (0.002) | 0.053  (0.002) | 0.019  (0.001) | 0.656  (0.003) | 0.349  (0.003) | 0.317  (0.002) |
| **Prs3** | 1.000  (0.000) | 1.000  (0.000) | 0.560  (0.001) | 1.000  (0.000) | 0.154  (0.001) | 1.000  (0.000) | 1.000  (0.000) | 0.127  (0.001) | 0.286  (0.001) | 1.000  (0.000) | 0.291  (0.001) |
| **Prs5** | 0.173  (0.001) | 0.011  (0.001) | 0.797  (0.002) | **0.001**  (0.000) | 0.480  (0.002) | 0.610  (0.003) | 0.346  (0.002) | **0.000**  (0.000) | 1.000  (0.000) | 0.376  (0.003) | 0.162  (0.004) |
| **Prb7** | 1.000  (0.000) | 0.470  (0.002) | 0.710  (0.002) | 0.101  (0.002) | 0.223  (0.002) | 1.000  (0.000) | 1.000  (0.000) | **0.001**  (0.000) | 0.040  (0.001) | 1.000  (0.000) | 1.000  (0.000) |

The sampled area codes are presented in Table 1. The test was developed with 10,000 randomizations. The bold values represent loci departed from Hardy-Weinberg equilibrium after Bonferroni correction (*P* < 0.0045); the standard errors (SEs) are indicated in parentheses.

**Table S6. Analysis to detect null alleles among the SSR markers evaluated in the areas sampled for *Prosopis ruscifolia.***

| **Marker** | **Null alleles** | | | | | | | | | | |
| --- | --- | --- | --- | --- | --- | --- | --- | --- | --- | --- | --- |
|  | **EPM** | **FQB** | **ROE** | **FFL** | **CJC** | **NSA** | **FSC** | **ECD** | **FRC1** | **FTR1** | **FTR2** |
| **Prs6** | 0.049 | 0.183 | 0.184 | 0.123 | 0.128 | 0.005 | 0.103 | 0.117 | 0.174 | 0.198 | **0.244** |
| **Prs1** | 0.000 | 0.069 | 0.000 | 0.023 | 0.026 | 0.013 | 0.000 | 0.105 | 0.000 | 0.000 | 0.000 |
| **Prb2** | 0.018 | 0.000 | 0.000 | 0.000 | 0.013 | 0.055 | 0.021 | 0.000 | 0.000 | 0.000 | 0.000 |
| **Prs2** | 0.000 | 0.082 | 0.041 | 0.122 | 0.073 | 0.012 | 0.083 | 0.114 | 0.032 | 0.102 | 0.014 |
| **Prb4** | 0.000 | 0.000 | 0.066 | 0.083 | 0.000 | 0.000 | 0.000 | 0.025 | 0.000 | 0.000 | 0.000 |
| **Prs7** | 0.081 | 0.184 | 0.114 | 0.053 | 0.171 | 0.185 | 0.092 | 0.113 | 0.172 | 0.140 | 0.144 |
| **Prs11** | **0.320** | 0.179 | 0.000 | 0.179 | 0.126 | 0.043 | 0.027 | 0.160 | **0.202** | 0.073 | 0.064 |
| **Prs12** | 0.000 | 0.166 | 0.022 | 0.038 | 0.013 | 0.000 | 0.000 | 0.114 | 0.000 | 0.008 | 0.000 |
| **Prs3** | 0.000 | 0.000 | 0.000 | 0.000 | 0.082 | 0.000 | 0.000 | 0.122 | 0.067 | 0.000 | 0.000 |
| **Prs5** | 0.100 | 0.095 | 0.000 | 0.000 | 0.000 | 0.000 | 0.000 | 0.169 | 0.000 | 0.007 | 0.030 |
| **Prb7** | 0.000 | 0.000 | 0.000 | 0.100 | 0.057 | 0.000 | 0.000 | 0.133 | 0.112 | 0.000 | 0.000 |
| **Average** | 0.05 | 0.09 | 0.04 | 0.07 | 0.06 | 0.03 | 0.03 | 0.11 | 0.07 | 0.05 | 0.05 |

The sampled area codes are presented in Table 1. The bold values represent loci with possible null alleles in each sampled area in which the null frequency was < 0.20.

**Table S7. Pairwise analysis of linkage disequilibrium in 11 SSR loci in *Prosopis ruscifolia* sampled from all areas.**

| **Loci pairwise** | ***P-*Value** | **Loci pairwise** | ***P-*Value** | **Loci pairwise** | ***P-*Value** |
| --- | --- | --- | --- | --- | --- |
| Prs6_&_Prs1 | **0.0000** | Prs7_&_Prs12 | 0.0356 | Prs12_&_Prb7 | 0.0070 |
| Prs6_&_Prb2 | 0.5333 | Prs11_&_Prs12 | 0.0727 | Prs3_&_Prb7 | 0.0614 |
| Prs1_&_Prb2 | 0.1562 | Prs6_&_Prs3 | 0.2043 | Prs5_&_Prb7 | 0.0022 |
| Prs6_&_Prs2 | 0.0179 | Prs1_&_Prs3 | 0.2200 | - | - |
| Prs1_&_Prs2 | **0.0000** | Prb2_&_Prs3 | 0.1220 | - | - |
| Prb2_&_Prs2 | 0.0046 | Prs2_&_Prs3 | 0.4121 | - | - |
| Prs6_&_Prb4 | **0.0000** | Prb4_&_Prs3 | 0.2120 | - | - |
| Prs1_&_Prb4 | **0.0000** | Prs7_&_Prs3 | 0.0460 | - | - |
| Prb2_&_Prb4 | **0.0000** | Prs11_&_Prs3 | 0.2031 | - | - |
| Prs2_&_Prb4 | **0.0000** | Prs12_&_Prs3 | 0.1772 | - | - |
| Prs6_&_Prs7 | **0.0000** | Prs6_&_Prs5 | 0.0433 | - | - |
| Prs1_&_Prs7 | **0.0000** | Prs1_&_Prs5 | 0.0376 | - | - |
| Prb2_&_Prs7 | 0.0203 | Prb2_&_Prs5 | 0.0005 | - | - |
| Prs2_&_Prs7 | **0.0000** | Prs2_&_Prs5 | 0.0477 | - | - |
| Prb4_&_Prs7 | **0.0000** | Prb4_&_Prs5 | 0.0130 | - | - |
| Prs6_&_Prs11 | **0.0000** | Prs7_&_Prs5 | 0.1011 | - | - |
| Prs1_&_Prs11 | 0.0460 | Prs11_&_Prs5 | 0.0844 | - | - |
| Prb2_&_Prs11 | 0.0014 | Prs12_&_Prs5 | 0.0016 | - | - |
| Prs2_&_Prs11 | **0.0000** | Prs3_&_Prs5 | 0.0159 | - | - |
| Prb4_&_Prs11 | **0.0000** | Prs6_&_Prb7 | 0.0004 | - | - |
| Prs7_&_Prs11 | **0.00016** | Prs1_&_Prb7 | 0.3613 | - | - |
| Prs6_&_Prs12 | 0.0070 | Prb2_&_Prb7 | 0.5438 | - | - |
| Prs1_&_Prs12 | 0.2004 | Prs2_&_Prb7 | 0.2418 | - | - |
| Prb2_&_Prs12 | 0.4423 | Prb4_&_Prb7 | 0.9319 | - | - |
| Prs2_&_Prs12 | 0.0853 | Prs7_&_Prb7 | **0.0000** | - | - |
| Prb4_&_Prs12 | 0.1102 | Prs11_&_Prb7 | 0.2116 | - | - |

The bold values suggest possible linkage disequilibrium for the pairwise-tested loci with *P* (1%) = 0.00018 after Bonferroni correction.

**Table S8. Pairwise analysis of linkage disequilibrium in 11 SSR loci in *Prosopis ruscifolia* excluding four sampled areas.**

| **Loci pairwise** | ***P-*Value** | **Loci pairwise** | ***P*-Value** |
| --- | --- | --- | --- |
| Prs6_&_Prs1 | 0.5599 | Prs6_&_Prs3 | 0.1777 |
| Prs6_&_Prb2 | 0.6486 | Prs1_&_Prs3 | 0.4363 |
| Prs1_&_Prb2 | 0.3582 | Prb2_&_Prs3 | 0.3428 |
| Prs6_&_Prs2 | 0.7701 | Prs2_&_Prs3 | 0.5406 |
| Prs1_&_Prs2 | 0.2542 | Prb4_&_Prs3 | 0.7482 |
| Prb2_&_Prs2 | 0.4325 | Prs7_&_Prs3 | 0.0356 |
| Prs6_&_Prb4 | 0.7006 | Prs11_&_Prs3 | 0.5032 |
| Prs1_&_Prb4 | 0.9133 | Prs12_&_Prs3 | 0.1943 |
| Prb2_&_Prb4 | 0.0093 | Prs6_&_Prs5 | 0.4627 |
| Prs2_&_Prb4 | 0.1574 | Prs1_&_Prs5 | 0.2001 |
| Prs6_&_Prs7 | 0.3493 | Prb2_&_Prs5 | 0.0006 |
| Prs1_&_Prs7 | 0.0029 | Prs2_&_Prs5 | 0.3531 |
| Prb2_&_Prs7 | 0.7020 | Prb4_&_Prs5 | 0.0191 |
| Prs2_&_Prs7 | 0.7739 | Prs7_&_Prs5 | 0.0711 |
| Prb4_&_Prs7 | 0.9643 | Prs11_&_Prs5 | 0.1278 |
| Prs6_&_Prs11 | 0.2283 | Prs12_&_Prs5 | 0.0751 |
| Prs1_&_Prs11 | 0.8805 | Prs3_&_Prs5 | 0.0180 |
| Prb2_&_Prs11 | 0.0735 | Prs6_&_Prb7 | 0.0637 |
| Prs2_&_Prs11 | 0.3828 | Prs1_&_Prb7 | 0.9915 |
| Prb4_&_Prs11 | 0.2269 | Prb2_&_Prb7 | 0.5684 |
| Prs7_&_Prs11 | 0.0876 | Prs2_&_Prb7 | 0.9458 |
| Prs6_&_Prs12 | 0.3314 | Prb4_&_Prb7 | 0.9410 |
| Prs1_&_Prs12 | 0.4318 | Prs7_&_Prb7 | 0.0942 |
| Prb2_&_Prs12 | 0.4151 | Prs11_&_Prb7 | 0.2697 |
| Prs2_&_Prs12 | 0.9856 | Prs12_&_Prb7 | 0.1485 |
| Prb4_&_Prs12 | 0.8476 | Prs3_&_Prb7 | 0.0244 |
| Prs7_&_Prs12 | 0.0190 | Prs5_&_Prb7 | 0.3704 |
| Prs11_&_Prs12 | 0.1448 | - | - |

Linkage disequilibrium was absent after Bonferroni correction (*P* = 0.00018, 1%) after excluding the Fazenda Quebracho Brasil, Chácara Jacaré, Fazenda Nsa. Sra. Aparecida and Carandazal station areas according to Fisher's exact probability test.

**Table S9.** *F*_ST_ calculated pairwise between all sampled areas of *Prosopis rubriflora* and *Prosopis ruscifolia* (Genepop v.1.2) and their respective geographic distances.

| ***Prosopis rubriflora*** | | | | | | | | | | | |
| --- | --- | --- | --- | --- | --- | --- | --- | --- | --- | --- | --- |
|  | AAL | FPT1 | FPT2 | FSC | RMS | FTR1 | FTR2 | FRC1 | FRC2 | FSM | FSV |
| AAL | X | 227 km | 228 km | 232 km | 218 km | 223 km | 231 km | 233 km | 234 km | 228 km | 262 km |
| FPT1 | 0.250 | X | 2 km | 18 km | 17 km | 33 km | 28 km | 5 km | 7 km | 12 km | 47 km |
| FPT2 | 0.205 | -0.008 | X | 16 km | 18 km | 31 km | 26 km | 4 km | 6 km | 14 km | 49 km |
| FSC | 0.251 | 0.027 | 0.032 | X | 33 km | 18 km | 11 km | 15 km | 13 km | 29 km | 61 km |
| RMS | 0.232 | 0.018 | 0.018 | 0.016 | X | 44 km | 42 km | 22 km | 24 km | 10 km | 49 km |
| FTR1 | 0.237 | 0.009 | 0.013 | 0.017 | 0.005 | X | 10 km | 32 km | 30 km | 45 km | 79 km |
| FTR2 | 0.241 | 0.016 | 0.018 | 0.009 | -0.002 | -0.008 | X | 26 km | 24 km | 40 km | 71 km |
| FRC1 | 0.234 | 0.003 | 0.003 | 0.033 | 0.023 | 0.011 | 0.016 | X | 3 km | 15 km | 47 km |
| FRC2 | 0.252 | -0.001 | 0.003 | 0.022 | 0.017 | 0.005 | 0.003 | 0.005 | X | 18 km | 48 km |
| FSM | 0.273 | 0.017 | 0.023 | 0.016 | 0.000 | -0.002 | -0.004 | 0.021 | 0.011 | X | 40 km |
| FSV | 0.256 | 0.040 | 0.039 | 0.025 | 0.004 | 0.016 | 0.014 | 0.063 | 0.029 | 0.014 | X |
| ***Prosopis ruscifolia*** | | | | | | | | | | | |
|  | EPM | FQB | ROE | FFL | CJR | NSA | FSC | ECD | FRC1 | FTR1 | FTR2 |
| EPM | X | 16 km | 23 km | 2 km | 3 km | 8 km | 17 km | 224 km | 12 km | 35 km | 26 km |
| FQB | 0.046 | X | 9 km | 14 km | 19 km | 24 km | 32 km | 240 km | 22 km | 51 km | 43 km |
| ROE | 0.056 | 0.027 | X | 22 km | 25 km | 28 km | 38 km | 244 km | 24 km | 56 km | 49 km |
| FFL | 0.035 | 0.025 | 0.027 | X | 5 km | 10 km | 19 km | 225 km | 15 km | 37 km | 28 km |
| CJR | 0.029 | 0.024 | 0.025 | 0.005 | X | 5 km | 15 km | 221 km | 12 km | 32 km | 24 km |
| NSA | 0.046 | 0.043 | 0.039 | 0.009 | 0.016 | X | 9 km | 217 km | 10 km | 28 km | 20 km |
| FSC | 0.051 | 0.031 | 0.035 | 0.022 | 0.011 | 0.036 | X | 207 km | 16 km | 18 km | 11 km |
| ECD | 0.101 | 0.058 | 0.067 | 0.040 | 0.071 | 0.071 | 0.082 | X | 220 km | 189 km | 198 km |
| FRC1 | 0.045 | 0.061 | 0.063 | 0.009 | 0.011 | 0.036 | 0.049 | 0.075 | X | 32 km | 26 km |
| FTR1 | 0.040 | 0.020 | 0.031 | 0.026 | 0.025 | 0.026 | 0.036 | 0.080 | 0.056 | X | 10 km |
| FTR2 | 0.079 | 0.054 | 0.047 | 0.031 | 0.029 | 0.049 | 0.037 | 0.100 | 0.053 | 0.51 | X |

**Note:** The sampled area codes for both *P. rubriflora* and *P. ruscifolia* are presented in Table 1.

The upper diagonal describes the geographic distances between the areas, and the lower diagonal shows the results of pairwise *F*_ST_ analysis. The blue cells represent the *F*_ST_ combinations with lower levels of population structure; the orange cells represent moderate differentiation between the areas; the red cells represent the pairwise values for areas with high levels of structure; and the dark red cells represent the pairwise values for areas with very high levels of genetic structure.
